# Discovery of a Potent and Selective TEAD Degrader with Durable Degradation Activity

**DOI:** 10.1002/advs.202503277

**Published:** 2025-10-05

**Authors:** Linhui Cao, Jing Yang, Yuhang Liu, Xiaotong Chen, Yufang Shi, Yunshuo Zhao, Yong Zhang, Jian Chen, Bowen Li, Wuqiang Wen, Lu Chen, Bo Peng, Lu Huang, Yanli Sun, Lixin Zhou, Matthew G Rees, Melissa M Ronan, Jennifer A Roth, Zhixiang Guo, Jing Xing, Guangya Zhu, Yazhou Wang, Baishan Jiang, Jing Lu, Kehao Zhao, Wenchao Lu

**Affiliations:** ^1^ School of Pharmacy Key Laboratory of Molecular Pharmacology and Drug Evaluation (Yantai University) Ministry of Education, China Collaborative Innovation Center of Advanced Drug Delivery System and Biotech Drugs in Universities of Shandong Yantai University Yantai 264005 China; ^2^ Lingang Laboratory Shanghai 200031 China; ^3^ Department of Hematology Tongji Hospital Frontier Science Center for Stem Cell Research Shanghai Key Laboratory of Signaling and Disease Research School of Life Sciences and Technology Tongji University Shanghai 200092 China; ^4^ Kygent Therapeutics Shanghai 201203 China; ^5^ Department of Radiation and Medical Oncology Medical Research Institute Frontier Science Center of Immunology and Metabolism Zhongnan Hospital of Wuhan University School of Pharmaceutical Sciences Wuhan University Wuhan 430071 China; ^6^ Department of Cardiovascular Surgery The First Affiliated Hospital of Anhui Medical University 218 Jixi Road Hefei 230022 China; ^7^ Broad Institute of MIT and Harvard Cambridge Massachusetts 02142 USA

**Keywords:** chemical probe, degrader, hippo pathway, TEAD, YAP

## Abstract

The TEA/ATSS (TEAD) family of transcription factors are key effectors of the Hippo pathway, exerting their function through interactions with the coactivators YAP and TAZ. Over the past five years, the development of YAP–TEAD disruptors has emerged as a central focus of both academic and industrial efforts aimed at targeting the Hippo pathway for cancer therapy. In this study, the discovery and comprehensive characterization of KG‐FP‐003, a potent, selective, and durable TEAD degrader is reported. KG‐FP‐003 exhibits superior activity compared to the lipid‐binding pocket (LBP) inhibitor MYF‐03‐176 and the TEAD–YAP protein–protein interaction (PPI) inhibitor IAG933, efficiently degrading all TEAD isoforms at low nanomolar concentrations in a ubiquitin–proteasome system (UPS)‐dependent manner. This degradation translates into more robust and sustained therapeutic responses both in vitro and in vivo. Furthermore, barcoded cell line screening revealed elevated sensitivity in several cancer types, including endometrial carcinoma, glioblastoma, ovarian epithelial tumors, and osteosarcoma. These findings position KG‐FP‐003 as a compelling lead candidate for TEAD isoform‐selective therapies and underscore its potential utility beyond Hippo‐dysregulated mesothelioma.

## Graphical abstract

KG‐FP‐003, a highly potent TEAD‐YAP PROTAC derived from the patented inhibitor is developed. It selectively degrades endogenous TEAD proteins in HiBiT systems without IMiD‐related off‐target effects. Screening across 867 cancer cell lines revealed broad and superior anti‐tumor activity, highlighting its therapeutic potential through targeted TEAD degradation.

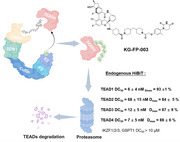

## 1 Introduction

Yes‐associated protein (YAP)/TEA/ATSS domain (TEADs) family proteins are evolutionarily conserved transcriptional complexes in Hippo pathway which play a pivotal role in stem cell maintenance, organ regeneration and cancer development.^[^ ^1^ ^]^ Genetic alteration of Hippo pathway components has been observed in malignant cancers. It was reported that 32% of mesothelioma patients has somatic *NF2* mutation and nearly 50% of meningiomas have allelic losses of *NF2*.^[^ ^2^, ^3^ ^]^ Pharmacological inhibition of TEADs by small molecules prevents growth of *NF2*‐null/*LATS1*‐fusion mesothelioma, schwannoma and meningioma.^[^ ^4^, ^5^ ^]^ *YAP1* amplification/fusion has been frequently found in solid tumors such as breast cancers and hepatocellular carcinomas.^[^ ^6^, ^7^ ^]^ Dysregulation of YAP/TEAD is also frequently correlated with chemoresistance to therapeutic drugs which shed light on combination therapy. Activation of YAP/TEAD established a senescence‐like tumor dormancy following combined EGFR/MEK inhibition in non‐small lung cancer cells.^[^ ^8^ ^]^ In ER‐positive breast cancer cells, loss of tumor suppressor FAT1 led to increased expression of CDK6 via YAP/TEAD signaling which contributes to resistance to CDK4/6 inhibitors.^[^ ^9^ ^]^ YAP/TEAD activation can also enable bypass of oncogenic KRAS in pancreatic cancers.^[^ ^10^ ^]^ Genome‐Wide CRISPR screens identified YAP/TEAD as synthetical lethal targets that can increase sensitivity of KRas^G12C^ inhibitors.^[^ ^11^ ^]^ Due to its significant role in tumorigenesis, tremendous efforts have been devoted to the development of TEAD inhibitors.^[^ ^12^, ^13^, ^14^, ^15^ ^]^ Although proof‐of‐concept studies demonstrating that TEAD transcription factors can be directly targeted by small molecules only emerged around 2015,^[^ ^16^ ^]^ multiple therapeutic strategies have already progressed to clinical evaluation. These include two palmitoylation inhibitors (VT3989, NCT04665206; IK930, NCT05228015), a TEAD–YAP protein–protein interaction (PPI) inhibitor (IAG933, NCT04857372), and an antisense oligonucleotide targeting YAP directly (ION537, NCT04659096).^[^ ^4^ ^]^

In the past decade, proteolysis‐targeting chimeras (PROTACs) have emerged as a powerful modality to eliminate disease‐associated proteins through degradation rather than inhibition .^[^ ^17^, ^18^, ^19^, ^20^ ^]^ While considerable progress has been made in developing TEAD inhibitors targeting palmitoylation, the potential of directly degrading TEAD proteins—particularly TEAD1, the isoform most strongly implicated in disease—remains underexplored. We hypothesize that TEAD degradation may offer broader therapeutic advantages over monotherapies directed at the lipid‐binding pocket .^[^ ^21^, ^22^ ^]^

Here, we report the development of KG‐FP‐003, a potent, selective, and durable PROTAC degrader that targets TEAD in vitro and in vivo. KG‐FP‐003 demonstrates single‐digit nanomolar potency, sustained activity for over 10 days in cultured cells, and exceptional selectivity across a wide panel of cell lines. Using barcoded PRISM screening across 867 cell lines, we evaluated the anti‐proliferative activity of KG‐FP‐003 and identified previously unrecognized cancer types that are more sensitive to TEAD degradation than to TEAD lipid‐binding pocket (LBP) or protein–protein interaction (PPI) inhibition. These findings highlight a promising new avenue for TEAD‐centric drug discovery and establish KG‐FP‐003 as a valuable addition to the toolkit of TEAD‐targeting chemical probes.

## 2 Results and Discussion

### 2.1 Development of PPI Inhibitor‐Based TEADs Protein Degrader

**Figure 1:**
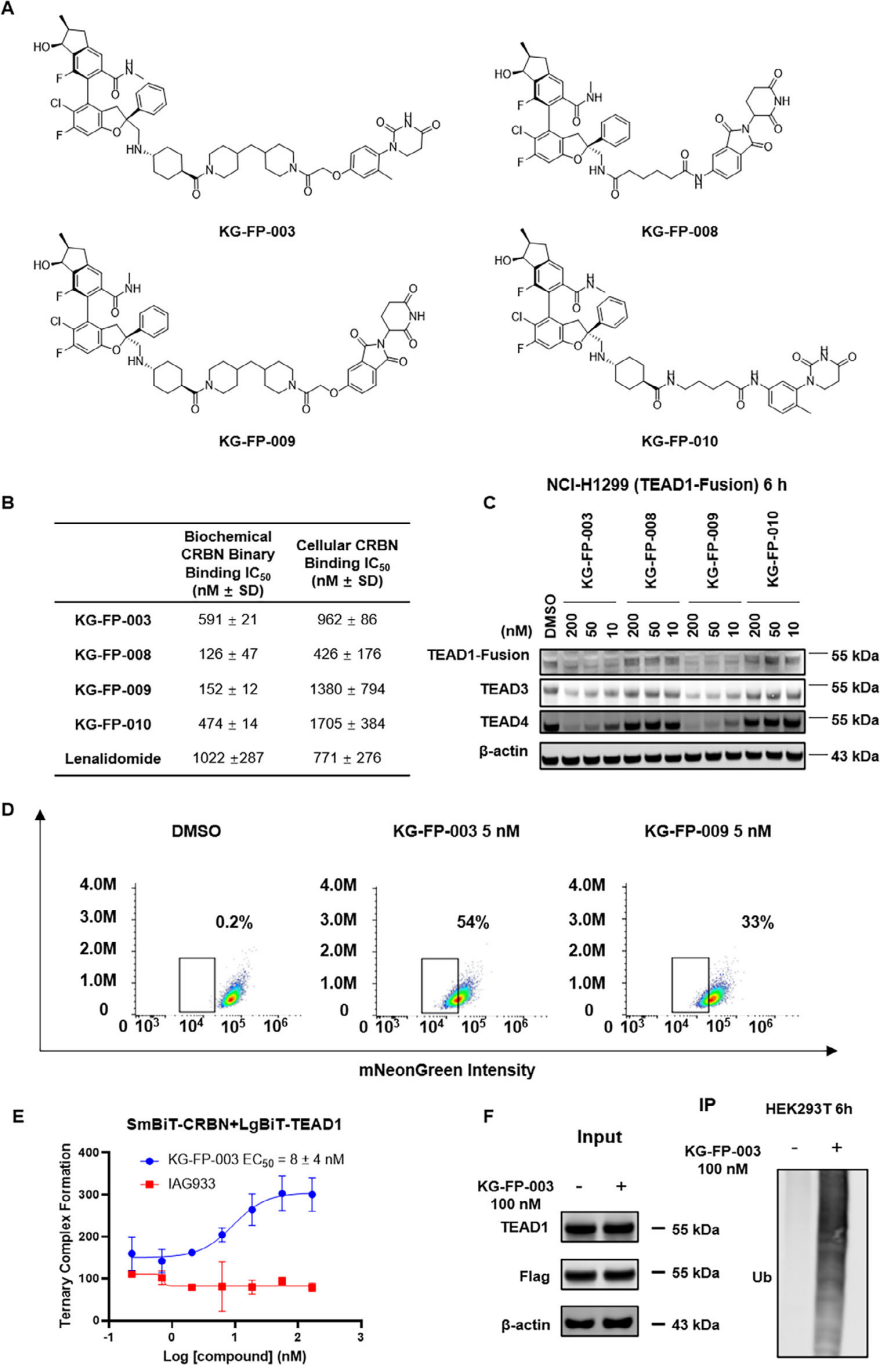
Design and Preliminary Screening of TEAD PROTACs. A) Chemical structures of TEAD PROTACs. B) CRBN binding affinity of TEAD PROTACs assessed through biochemical and cellular engagement assays in biological triplicates. Data are representative of three independent experiments. C) Immunoblot analysis of TEAD1 and TEAD4 protein levels in TEAD1‐mNeonGreen knock‐in NCI‐H1299 cells treated with degraders for 6 h at the indicated concentrations. D) Flow cytometry analysis of TEAD1 degradation in TEAD1‐mNeonGreen NCI‐H1299 cells treated with degraders at 5 nM for 24 h. Data are representative of three independent experiments. E) KG‐FP‐003 induces ternary complex formation between CRBN (41‐442) and TEAD1‐YBD (208‐424) in HEK293T cells. F) Immunoprecipitation (IP) analysis was performed in HEK293T cells transiently co‐transfected with Flag‐TEAD1 and HA‐Ub plasmids (1:1 ratio). Cells were pre‐treated with 400 nM bortezomib for 2 h to block proteasome‐mediated degradation, followed by treatment with 100 nM KG‐FP‐003 for 6 h. Data shown are representative of three independent experiments.

As an initial effort, based on the reported binding pose of the YAP/TEAD PPI inhibitor (PDB ID: 8P0M),^[^ ^4^ ^]^ we undertook the design and synthesis of a series of TEAD PROTACs by refining the physicochemical attributes of the warhead, linker, and CRBN ligand. This effort was guided by a comprehensive analysis of structural features observed in our disclosed TEAD‐YAP warheads (CN116217554B) (**Figure** **1**A). To assess whether the synthesized PROTACs retain the ability to bind cereblon (CRBN), we performed homogeneous time‐resolved fluorescence (HTRF) assays using recombinant CRBN‐DDB1 and TEAD‐YBD proteins, as well as NanoLuc‐CRBN‐overexpressing HEK293T cells, with lenalidomide and IAG933 as controls (Figure 1B; Figure S1, Supporting Information). Among the synthesized compounds, KG‐FP‐003, KG‐FP‐008, KG‐FP‐009, and KG‐FP‐010 exhibited slightly enhanced CRBN binding in vitro compared to lenalidomide. However, their binding in cells was relatively weaker, likely due to the intrinsic permeability limitations of PROTAC molecules (Figure 1B). To better assess their degradation efficiency,  we generated TEAD1‐mNeonGreen fusion NCI‐H1299 cells, and the degradation activity of these compounds was initially evaluated via immunoblot analysis (Figure 1C). Among the tested PROTACs, KG‐FP‐003 and KG‐FP‐009 demonstrated the most potent degradation activity, achieving significant target degradation at a concentration of 10 nM, which was also confirmed by measuring intracellular mNeonGreen fluorescence intensity by flow cytometry (Figure 1D). Treatment with 5 nM KG‐FP‐003 demonstrated degradation activity comparable to, yet slightly superior to KG‐FP‐009.

To confirm ternary complex formation in cells, we generated SmBiT‐CRBN and LgBiT‐TEAD1 fusion constructs and established a bioluminescence‐based complementation assay. KG‐FP‐003 treatment robustly induced formation of the TEAD1–CRBN ternary complex, with a half‐maximal effective concentration (EC_50_) of 8 ± 4 nM. To identify critical residues involved in this interaction, we performed computational modeling of the ternary complex using ClusPro 2.0^[^ ^23^ ^]^ and Surflex‐Dock Geom (Sybyl‐X 2.1.1; Tripos, Inc.). Site‐directed mutagenesis of key interface residues—TEAD1‐E417W and CRBN‐P352W, H353W, and S375W—abolished the bioluminescence signal, indicating disruption of ternary complex formation. These results not only confirm the specificity of KG‐FP‐003‐mediated target engagement but also functionally validate the predicted binding mode (Figure 1E; Figure S2, Supporting Information). To determine whether the compound‐induced degradation occurs through the ubiquitin–proteasome system (UPS), we transiently transfected HEK293T cells with Flag‐TEAD1 and HA‐Ub plasmids. Immunoprecipitation (IP) assays revealed that treatment with the compound markedly increased TEAD1 ubiquitination, indicating that the compound promotes UPS‐dependent degradation of TEAD1^[^ ^24^ ^]^ (Figure 1F).

### 2.2 MOA Characterization of KG‐FP‐003

**Figure 2:**
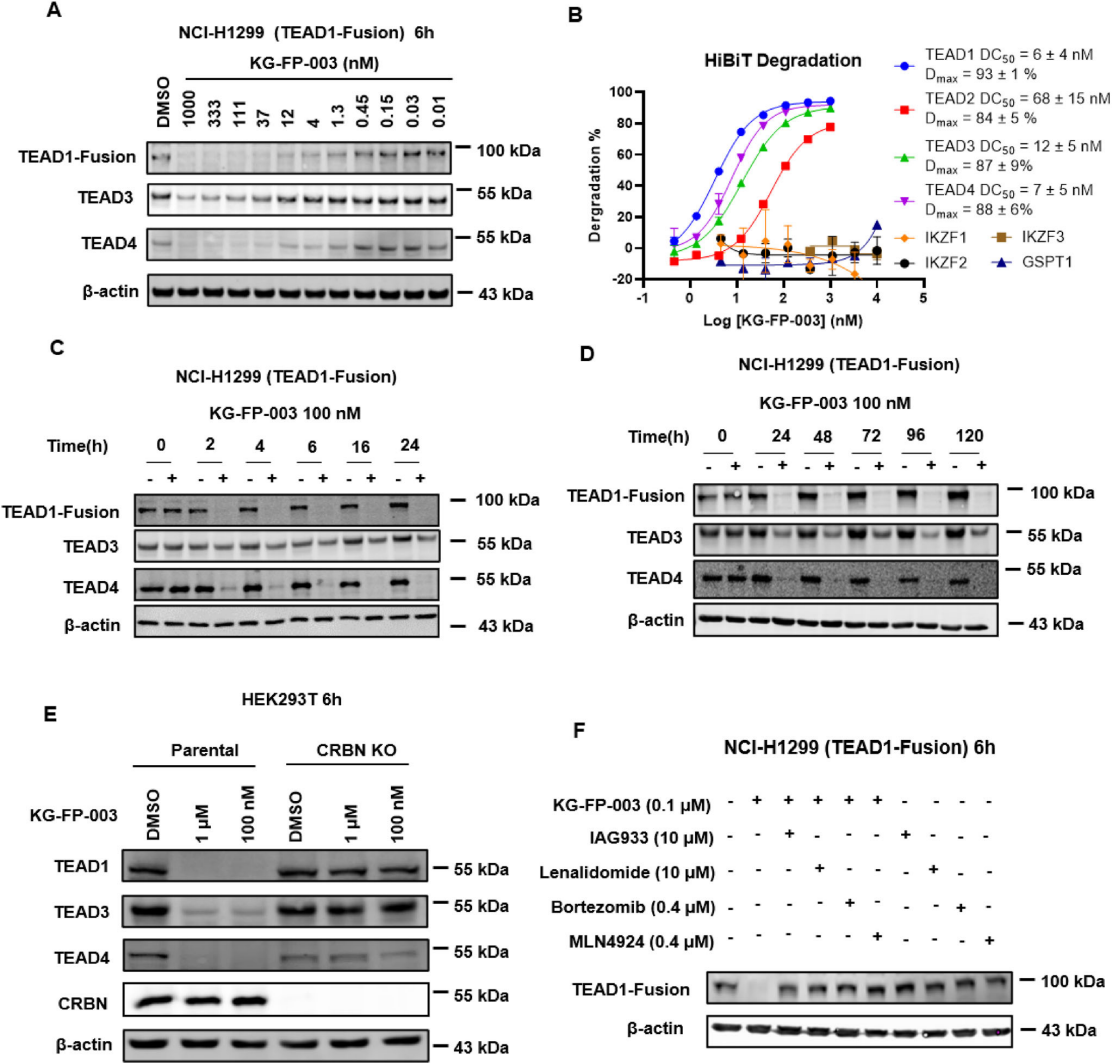
Characterization of KG‐FP‐003. A) KG‐FP‐003 induces dose‐dependent degradation of TEAD proteins in TEAD1‐mNeonGreen fusion NCI‐H1299 cells after 6 h treatment. B) Degradation efficacy and off‐target assessment of KG‐FP‐003 across various HiBiT‐tagged cell lines in biological triplicates. KG‐FP‐003 were treated at indicated concentrations for 6 h. Data are representative of three independent experiments. C,D) Degradation kinetics of TEAD1/3/4 by KG‐FP‐003 in cells treated with 100 nM KG‐FP‐003. E) Western blot analysis of TEAD1/3/4 degradation in wild‐type and CRBN‐knockout HEK293T cells following 6 h treatment with KG‐FP‐003. F) Rescue experiment of TEAD1 degradation in HEK293T cells pretreated with inhibitors at indicated concentrations for 1 h. Data are representative of three independent experiments.

To further investigate the degradation profile of KG‐FP‐003, we performed dose‐response analyses using immunoblots in TEAD1‐mNeonGreen fusion NCI‐H1299 cells. KG‐FP‐003 showed potent degradation activity against TEAD1, TEAD3, and TEAD4, with all DC_50_ values in the nanomolar range (**Figure** **2**A). Due to the lack of validated antibodies for all TEAD isoforms—particularly TEAD2—we generated endogenous HiBiT‐tagged TEAD1–4 cell lines via CRISPR technology to enable comprehensive isoform detection.^[^ ^25^ ^]^ HiBiT assays demonstrated efficient degradation of all TEAD isoforms by KG‐FP‐003 at nanomolar concentrations (TEAD1 DC_50_ = 6 ± 4 nM, TEAD2 DC_50_ = 68 ± 15 nM, TEAD3 DC_50_ = 12 ± 5 nM, TEAD4 DC_50_ = 7 ± 5 nM), significantly outperforming lipid‐binding pocket (LBP)‐based TEAD PROTACs^[^ ^21^ ^]^ (Figure S3, Supporting Information). Importantly, no off‐target degradation was detected among known IMiD substrates such as IKZF1‐3 or the confounding protein GSPT1^[^ ^26^ ^]^ (Figure 2B). To validate the physiological relevance of these findings, Western blot analyses were performed in HEK293T‐HiBiT cells, confirming that TEAD degradation indeed closely mirrored those observed in the luminescence‐based HiBiT reporter assays (Figure S4, Supporting Information). We note that unlike other TEAD isoforms, HiBiT‐TEAD2 could not be detected using the anti‐HiBiT antibody under the same condition, likely due to the relatively low expression of TEAD2 in HEK293T cells^[^ ^27^ ^]^ (Figure S5,Supporting Information).

To further understand the kinetics of KG‐FP‐003–induced TEAD degradation, we conducted a time‐course study in TEAD1‐mNeonGreen fusion NCI‐H1299 cells. KG‐FP‐003 triggered rapid, time‐dependent degradation of TEAD1, achieving near‐complete depletion within 2 h, with the effect sustained for up to 12 days (Figure 2C,D; Figure S6, Supporting Information). This degradation was minimal in CRBN‐null HEK293T cells (Figure 2E). Moreover, pretreatment with the TEAD/YAP inhibitor IAG933, lenalidomide, the neddylation inhibitor MLN4924, or the proteasome inhibitor bortezomib effectively blocked KG‐FP‐003–mediated degradation, confirming that this process is CRBN‐dependent and relies on the CUL4‐CRBN ubiquitin–proteasome system (Figure 2F).

### 2.3 KG‐FP‐003 Demonstrates Excellent Selectivity and Anti‐Tumor Activity in Mesothelioma Model

**Figure 3:**
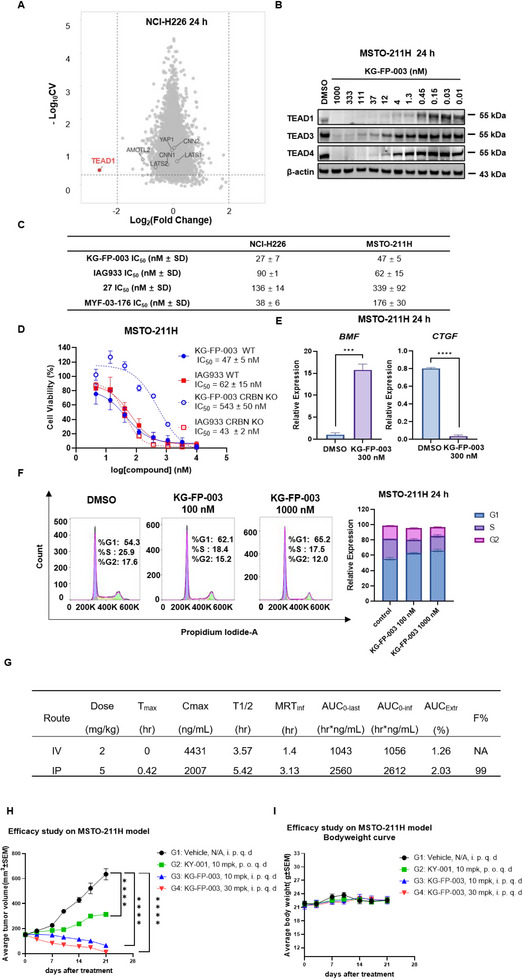
Characterization of KG‐FP‐003 efficacy in mesothelioma cells. A) Proteomic analysis of NCI‐H226 cells following 24 h treatment with 100 nM KG‐FP‐003 with biological triplicates. B) Western blot analysis of MSTO‐211H cells treated with various doses of KG‐FP‐003 and IAG933 for 24 h. Data are representative of three independent experiments. C) Comparison of antiproliferative activities of TEAD inhibitors or degraders in NCI‐H226 and MSTO‐211H cells in 3‐day antiproliferation experiments in biological triplicates. Data are representative of three independent experiments. D) Antiproliferative effects of KG‐FP‐003 and IAG933 in wild‐type and CRBN‐knockout MSTO‐211H cells in biological triplicates. Data are representative of three independent experiments. E) Downstream gene expression changes in MSTO‐211H cells following KG‐FP‐003 treatment. Data are representative of three independent experiments. F) Cell cycle analysis of KG‐FP‐003 in MSTO‐211H cells, showing its effect on cell cycle progression based on three biological replicates. Data are representative of three independent experiments. G) Pharmacokinetic profile of KG‐FP‐003 following intravenous (2 mg kg^−1^) and intraperitoneal (5 mg kg^−1^) administration in ICR male mice (*n*  =  3 per group). H) Tumor growth inhibition in MSTO‐211H mesothelioma xenograft models treated with KG‐FP‐003 (10 or 30 mg kg^−1^, i.p., q.d.), KY‐001 (10 mg kg^−1^, p.o., q.d.), or vehicle control for 21 days (*n*  =  6 per group). I) Body weight changes in MSTO‐211H xenograft mice model during the treatment period (*n*  =  6 per group).

Given that dysregulation of the Hippo signaling pathway is a hallmark of malignant pleural mesothelioma, we evaluated the degradation efficacy of KG‐FP‐003 in the well‐established mesothelioma cell line NCI‐H226, a classical model for TEAD inhibitor characterization. Cells were treated with 100 nM KG‐FP‐003 for 24 h, followed by multiplexed, mass spectrometry‐based proteomics analysis. The results revealed selective downregulation of TEAD1 and TEAD4, consistent with immunoblotting data (**Figure** **3**A,B; Figures S7 and S8, Supporting Information). This degradation translated into potent antiproliferative activity, with IC_50_ values of 27  ± 7 nM in NCI‐H226 cells and 47 ± 5 nM in MSTO‐211H cells. Notably, KG‐FP‐003 outperformed the PPI inhibitor IAG933, the LBP inhibitor MYF‐03‐176,^[^ ^28^ ^]^ and the previously reported LBP‐based TEAD PROTAC 27^[^ ^21^ ^]^ (Figure 3C; Figure S9, Supporting Information). To confirm the requirement for CRBN‐mediated degradation, we generated CRBN‐knockout MSTO‐211H cells. Loss of CRBN significantly impaired KG‐FP‐003′s antiproliferative activity, while the activity of IAG933 remained unaffected (Figure 3D), confirming the degrader's CRBN‐dependent mechanism of action. In parallel, qPCR analysis in MSTO‐211H cells revealed dramatic upregulation of the pro‐apoptotic gene *BMF* and downregulation of the TEAD target gene *CTGF*, supporting functional consequences of TEAD degradation (Figure 3E). Additionally, flow cytometry analysis demonstrated that KG‐FP‐003 induced G1 phase cell cycle arrest in a dose‐dependent manner, further highlighting its antiproliferative mechanism of action (Figure 3F).

To evaluate in vivo efficacy, we first assessed the pharmacokinetic (PK) profile of KG‐FP‐003 in ICR male mice. Intraperitoneal (i.p.) administration at 5 mg kg^−1^ resulted in rapid absorption (T_max_ = 0.42 h), a high peak plasma concentration (C_max_ = 2007 ng mL^−1^), a favorable half‐life (t_1/2_ = 5.42 h), and excellent bioavailability (99%) (Figure 3G). These pharmacokinetic properties provided a solid foundation for subsequent in vivo efficacy studies. Administration of KG‐FP‐003 at a lower dose of 3 mg kg^−1^ i.p. in an MSTO‐211H pleural mesothelioma xenograft model led to pronounced degradation of TEAD1 protein and significant tumor growth inhibition compared to vehicle‐treated controls, indicating a robust on‐target effect (Figure S10, Supporting Information).

Building on these results, we further evaluated KG‐FP‐003 in the *NF2*‐deficient MSTO‐211H xenograft model, which is considered the gold standard for TEAD/YAP‐targeting agents. KG‐FP‐003 was administered intraperitoneally at doses of 10 and 30 mg kg^−1^ to BALB/c nude mice bearing subcutaneous tumors. The TEAD/YAP warhead KY‐001 was used as a positive control. During the 21‐day treatment period, KG‐FP‐003 demonstrated potent dose‐dependent antitumor activity. Specifically, KY‐001 achieved a tumor growth inhibition (TGI) rate of 66% at 10 mg kg^−1^, while KG‐FP‐003 exhibited significantly higher efficacy, with TGI reaching 118% at 10 mg kg^−1^ and increasing to 129% at 30 mg kg^−1^ (Figure 3H). Importantly, all treated mice maintained stable body weights throughout (Figure 3I), indicating good tolerability. To further characterize safety, we conducted a 7‐day toxicity assessment in MSTO‐211H tumor‐bearing mice treated daily with 10 or 30 mg kg^−1^ of KG‐FP‐003 (i.p.). Hematological analyses showed no significant deviations from baseline, and all values remained within physiological ranges. Histopathological examination of major organs—including the heart, liver, spleen, lungs, and kidneys—revealed no structural abnormalities or pathological lesions (Figures S11–S14, Supporting Information), supporting the favorable safety profile of KG‐FP‐003 at the tested doses.

### 2.4 PRISM Screen of KG‐FP‐003 in 867 Cancer Cell Lines

**Figure 4:**
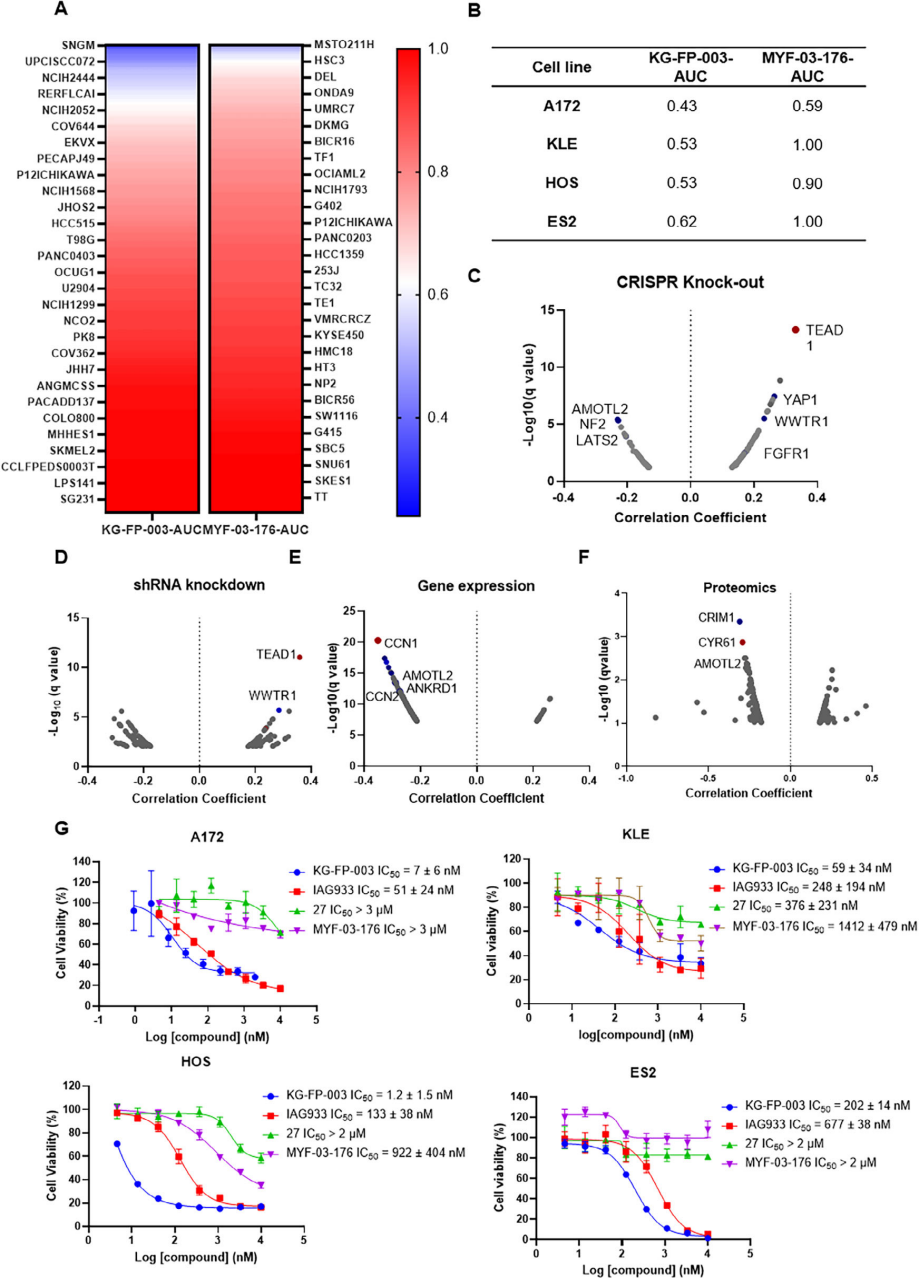
PRISM‐based Functional Genomic Profiling and Validation of KG‐FP‐003 Sensitivity. A) Comparison of area under the curve (AUC) values between KG‐FP‐003 and the TEAD palmitoylation inhibitor MYF‐03‐176 across 867 cancer cell lines in the PRISM screening platform. B) AUC values of representative cancer cell lines identified as highly sensitive to KG‐FP‐003. C,D) Correlation analysis between KG‐FP‐003 sensitivity and gene dependency profiles derived from genome‐wide CRISPR knockout (C) and shRNA knockdown (D) datasets. E,F) Association between KG‐FP‐003 sensitivity and baseline gene expression (E) or global proteomics (F) across the PRISM cell line panel. G) Validation of antiproliferative activity of KG‐FP‐003 in selected sensitive cell lines following 3‐day compound treatment. Data are representative of three independent experiments.

Given the relatively limited therapeutic scope of TEAD lipid‐binding pocket (LBP) inhibitors as monotherapies, we sought to delineate the pharmacological profile of KG‐FP‐003 by leveraging the PRISM assay across a diverse panel of 867 cancer cell lines. Cells were treated with varying concentrations of KG‐FP‐003 for five days, after which viable cells were collected and analyzed via barcode sequencing. Notably, when we compared the area under the curve (AUC) between KG‐FP‐003 and MYF‐03‐176 (a representative TEAD LBP inhibitor), we identified several cancer cell lineages—such as the glioblastoma cell line A172, the endometrial carcinoma cell line KLE, the osteosarcoma cell line HOS, and the ovarian carcinoma cell line ES2—that exhibited pronounced sensitivity to KG‐FP‐003 but not to MYF‐03‐176 (**Figure** **4**A,B; Figure S15, Supporting Information). Further analysis of the PRISM dataset revealed that the overall antiproliferative profile of KG‐FP‐003 most closely resembled that of TEAD1 dependency observed in CRISPR knockout and shRNA knockdown screens (Figure 4C,D). In addition, pathway enrichment analyses highlighted strong correlations with known TEAD‐associated genes, including *WWTR1*, *YAP1*, and *FGFR1*, as well as negative regulators of the Hippo pathway such as *NF2*, *LATS2*, and *AMOTL2*—supporting a clear on‐target mechanism of action. Transcriptomic and proteomic profiling from the PRISM dataset further demonstrated strong associations between KG‐FP‐003 sensitivity and expression of canonical YAP/TEAD downstream targets such as *CCN1*, *CCN2*, and *ANKRD1* (Figure 4E,F).

To validate the PRISM‐based predictions, we assessed the antiproliferative activity of KG‐FP‐003 in a panel of sensitive cell lines using CellTiter‐Glo (CTG) assays. Across all tested lines, KG‐FP‐003 consistently outperformed multiple benchmark TEAD‐targeting agents, including MYF‐03‐176 (an LBP inhibitor), IAG933 (a PPI inhibitor), compound 27 (an LBP‐based PROTAC), and the analog KG‐FP‐009, thereby demonstrating a broader and more potent antiproliferative profile (Figure 4G; Figure S16, Supporting Information). Given the ease of establishing xenograft models using ES2 cells and the clinical relevance of this model—characterized by elevated expression of TEAD target genes *CYR61* and *ANKRD1*, both associated with poor prognosis in TCGA datasets (Figures S17 and S18, Supporting Information). We confirmed compound‐induced degradation of TEAD1—the most disease‐relevant TEAD isoform as indicated by DepMap—in ES2 cells, and subsequently evaluated the efficacy of KG‐FP‐003 in this context (Figure S19, Supporting Information).Treatment with KG‐FP‐003 at doses of 10 and 30 mg kg^−1^ led to significant tumor regression, although the magnitude of response was less robust compared to the MSTO‐211H model, highlighting MSTO‐211H as a more responsive and thus more suitable model for evaluating TEAD‐targeting agents (Figure S20, Supporting Information).

### 2.5 Evaluation and Mechanistic Exploration of KG‐FP‐003 in Glioblastoma

Building upon screening results from the PRISM database, we selected the glioblastoma multiforme (GBM) cell line A172—representing one of the most aggressive forms of primary brain tumors—for in‐depth mechanistic studies. Proteomic profiling revealed that, consistent with findings in mesothelioma models, KG‐FP‐003 significantly and selectively downregulated TEAD1 protein levels in A172 cells (**Figure** **5**A; Figure S21, Supporting Information). Notably, KG‐FP‐003 also induced upregulation of TAZ protein, potentially via a compensatory feedback mechanism (Figure S22, Supporting Information). Interestingly, both KG‐FP‐003 and the PPI inhibitor IAG933 increased TAZ protein expression after 24 h treatment in A172 cells, as confirmed by immunoblotting, suggesting that this effect may result from PPI inhibition rather than degradation alone.

Figure 5Modulation of TEAD signaling and mechanistic investigation of KG‐FP‐003 activity in A172 glioblastoma cells. A) Quantitative proteomic analysis of A172 cells treated with 100 nM KG‐FP‐003 for 24 h, showing selective downregulation of TEAD1. B) Immunoblot analysis demonstrating dose‐dependent degradation of TEAD1/3 and suppression of TEAD target genes (*CYR61*, *PTX3*) following 24 h KG‐FP‐003 treatment. C) qPCR analysis was performed to assess the expression of canonical TEAD downstream genes (*CTGF*, *ANKRD1*, *NPPB*, and *PTX3*) in A172 cells following treatment with KG‐FP‐003 or IAG933 for 24 h. For washout experiments, cells were treated with KG‐FP‐003 or IAG933 for 6 h, washed three times with PBS, and then incubated in fresh complete medium for an additional 18 h before RNA collection. Data represent biological triplicates. D) Number of DEGs identified via RNA‐seq in A172 cells treated with 30 nM KG‐FP‐003 or IAG933 for 24 h. E) Venn diagram showing overlap between DEGs from KG‐FP‐003– versus DMSO–treated cells and IAG933– versus DMSO–treated cells. F) Volcano plot analysis of DEGs in KG‐FP‐003–treated groups relative to IAG933–treated groups; selected TEAD target genes are highlighted. G) KEGG pathway enrichment analysis comparing transcriptional changes induced by KG‐FP‐003 versus IAG933. H) Gene Set Enrichment Analysis illustrating pathways significantly enriched in KG‐FP‐003–treated cells relative to IAG933, including Hippo signaling and cell cycle regulation. Data are representative of three independent experiments.

**Figure:**
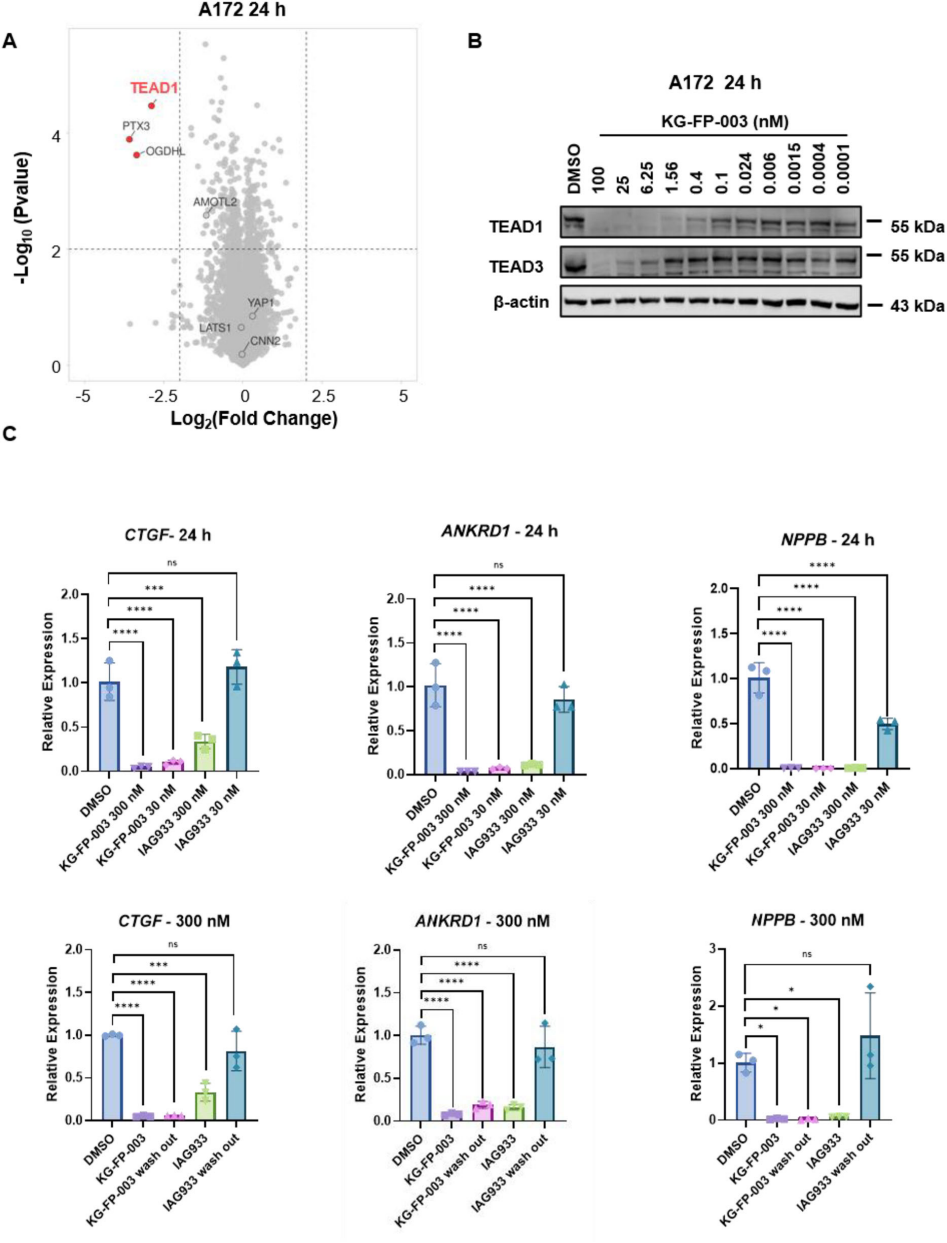

**Figure:**
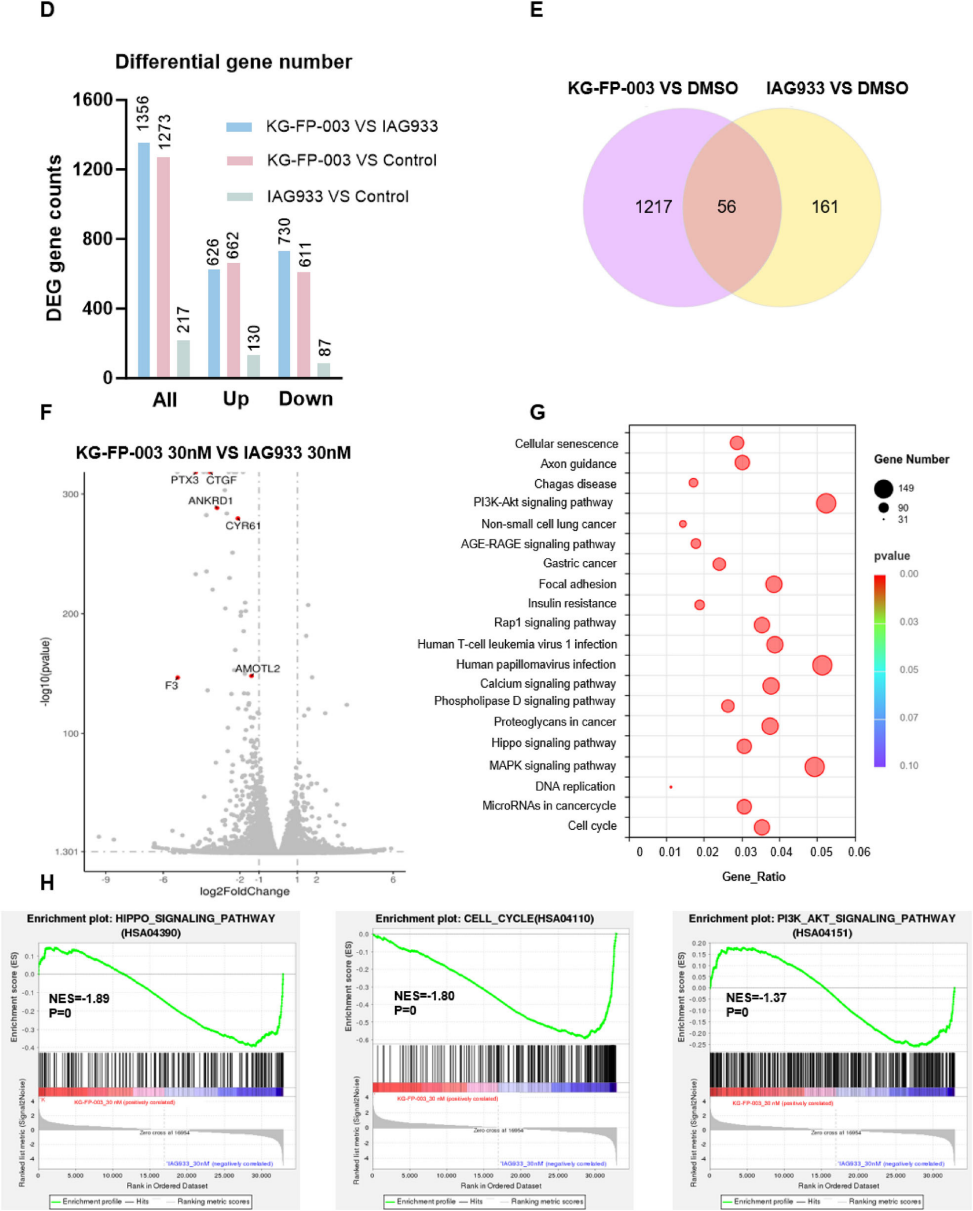

Immunoblot analyses further demonstrated that KG‐FP‐003 not only achieved complete degradation of TEAD1 and TEAD3 but also downregulated the expression of downstream TEAD target genes, including *CYR61* and *PTX3*, in a dose‐dependent manner—exhibiting more potent inhibitory effects than IAG933 (Figure 5B; Figure S23, Supporting Information). Consistently, qPCR assays revealed that even at low concentrations, KG‐FP‐003 induced a more substantial reduction in the expression of *CTGF*, *ANKRD1*, and *NPPB* compared to IAG933. In washout experiments, KG‐FP‐003 elicited sustained and pronounced repression of these genes, including *PTX3*, whereas the effects of IAG933 were significantly diminished following compound removal (Figure 5C; Figure S24, Supporting Information). These data from wash‐out studies highlight the superior and sustained TEAD‐targeting activity of the PROTAC molecule KG‐FP‐003.

To further delineate the broader transcriptional consequences of TEAD degradation, we treated A172 cells with 30 nM KG‐FP‐003 or the PPI inhibitor IAG933 for 24 h, followed by RNA sequencing‐based transcriptomic profiling. At this relatively low concentration, KG‐FP‐003 elicited a substantially more pronounced transcriptional response compared to IAG933, resulting in 662 upregulated and 611 downregulated genes. In contrast, IAG933 treatment yielded only 217 differentially expressed genes (DEGs) (Figure 5D). Comparative analysis revealed a modest overlap of 56 genes between the two treatment groups, accounting for 25.8% of IAG933‐regulated genes but only 4% of those affected by KG‐FP‐003 (Figure 5E). Volcano plot analysis further highlighted significant downregulation of canonical TEAD target genes—including *PTX3*, *CTGF*, *ANKRD1*, *CYR61*, and *AMOTL2*—each exhibiting ≥2‐fold changes and adjusted *p*‐values ≤ 0.05 (Figure 5F), consistent with proteomic and qPCR validation. Pathway‐level analysis using KEGG enrichment and Gene Set Enrichment Analysis (GSEA) demonstrated that KG‐FP‐003 induced more extensive disruption of Hippo signaling and cell cycle regulatory pathways than IAG933 (Figure 5G,H), further substantiating the mechanistic basis and superior efficacy of TEAD‐targeted protein degradation.

## 3 Conclusion

Given their aberrant activation and pivotal role in numerous cancers, TEAD transcription factors have emerged as highly promising therapeutic targets in oncology. However, drug discovery efforts have predominantly focused on the development of TEAD inhibitors, while targeted degradation strategies for TEADs remain largely underexplored. Progress in this area has been further hampered by the limited availability of subtype‐specific TEAD antibodies, restricting most evaluations to ectopic expression systems. In this study, we present KG‐FP‐003 as a potent, selective, and durable TEAD degrader. To enable precise and quantitative assessment of endogenous TEAD protein levels, we employed CRISPR/Cas9‐mediated genome editing to generate HEK293T cell lines stably expressing HiBiT‐tagged TEAD1–4. In this system, KG‐FP‐003 demonstrated robust degradation activity, achieving ≈90% reduction in TEAD1/3/4 and over 80% reduction in TEAD2 protein levels. Importantly, TEAD degradation translated into potent anti‐proliferative effects in mesothelioma cells. Moreover, PRISM profiling revealed that TEAD degraders display a distinct sensitivity pattern across cancer types, identifying additional tumor models beyond pleural mesothelioma that are responsive to TEAD degradation but not to TEAD lipid‐binding domain (LBD) inhibition. In summary, our findings establish KG‐FP‐003 as a promising best‐in‐class TEAD degrader with broad therapeutic potential. Its potent degradation efficacy, durable activity, and ability to suppress tumor growth across multiple cancer models provide a strong foundation for further preclinical development.

## 4 Experimental Section

### Chemistry

The Supporting Information contains comprehensive descriptions of the synthetic procedures along with complete characterization data for the final compounds.

### Cell Culture

Cells were cultured in a humidified incubator at 37 °C with 5% CO_2_. HEK293T cells (Meilunbio, #CL0169), A172 cells (Aoruicell, #ORC0012), and CAOV3 cells (Aoruicell, #ORC0456) were cultured in DMEM (Meilunbio, #MA0212) supplemented with 10% FBS and 1% Pen/Strep. NCI‐H226 cells (Meilunbio, #CL0026) and MSTO‐211H cells (Fuheng Bio, #FH1284) were cultured in RPMI‐1640 (Meilunbio, #MA0546) with 10% FBS and 1% Pen/Strep. HOS cells (Aoruicell, #ORC0012) were cultured in MEM (Meilunbio, #MA0217) with 10% FBS and 1% Pen/Strep. KLE cells (Aoruicell, #ORC0110) were cultured in DMEM/F12 (Meilunbio, #MA0214) with 10% FBS and 1% Pen/Strep. MSTO‐211H CRBN knockout cells were generated from wild‐type MSTO‐211H cells. HEK293T‐NanoLuc‐CRBN‐overexexpressing cells were generated from wild‐type HEK293T cells. HEK293T CRBN knockout cells were kindly provided by Prof. Yong Cang (ShanghaiTech University). All cell lines were authenticated by STR analysis and tested negative for mycoplasma.

### CRBN Binding Assay‐*Biochemical Binding*

The HTRF system consisted of the following components: 5 µL of CRBN‐ DDB1ΔBPB protein solution (final concentration 10 nM), 5 µL of fluorescently labeled lenalidomide analogue solution (final concentration 200 nM), and 10 µL of HTRF anti‐His‐Tb Conjugate solution, diluted 400‐fold with assay buffer (Revvity, #61HI2TLF). These components were mixed thoroughly to form a 20 µL reaction system, which was then added to a HTRF 384‐well low‐volume plate (Taidu Biotech, #GC1238). Next, 400 nL of compound solution (initial concentration 10 µM, followed by a three‐fold gradient dilution) was dispensed into the wells using the positive‐pressure‐based I‐DOT dispenser. After 1 h of incubation at room temperature, the HTRF signal was measured using an Envision plate reader (Revvity). Finally, the data were analyzed using non‐linear regression curves in GraphPad Prism 9.5 software.

### Intracellular Binding

NanoLuc‐CRBN‐overexpressing HEK293T were seeded in 96‐well plates at a density of 2000 per well. The following day, compounds was added to the plate starting from 10 µM for a 3‐fold gradient dilution and incubated for 2 h. Lenalidomide was used as a control. Afterwards, 500 nM fluorescently labelled lenalidomide analogue was added to each well, and incubated for another 2 h, the Nano‐Light Luciferase Reporter Assay Kit (Meilunbio, #MA0521‐2) was added, with 25 µL per well. Finally, the signal was measured using an Envision plate reader (Revvity) and the data were analyzed using the GraphPad Prism 9.5 software.

### NanoBiT Recruitment Assay

*CRBN* was cloned into the SmBiT Flexi vector pFN35K (Promega), while *TEAD1* was cloned into the Promega LgBiT Flexi vector pFN33K (Promega). A total of 8 µg of plasmid DNA, consisting of 2 µg of SmBiT‐CRBN plasmid and 6 µg of LgBiT‐TEAD1 plasmid, was mixed with 24 µL of the liposome transfection reagent Lipo2000 (Meilunbio, #MA0672), added to 300 µL of Opti‐MEM Reduced Serum medium (Meilunbio, #PWL042), and incubated for 20 min at room temperature. The resulting mixture was then added to 3 mL of HEK293T cell suspension at a density of 1 × 10^6^ cells mL^−1^ in DMEM medium containing 1% FBS, mixed thoroughly, and 18.4 µL of the mixture was transferred to each well of a white 384‐well plate (Corning, #3570), followed by incubation for 24 h. Subsequently, 2.5 µL of MLN4924 (MCE, #HY‐70062) was added to reach a final concentration of 1 µM, and the cells were incubated for an additional 4 h. Next, 3 µL of compound KG‐FP‐003 at the indicated concentrations was added, and incubation continued for 3 more hours. Afterward, a mixture of Nano‐Glo Live Cell Substrate and Nano‐Glo LCS Dilution Buffer (Promega, #N2011) was added to the 384‐well plates at 6 µL per well and incubated for 20 min at room temperature. Finally, the luminescence signal was measured using an Envision plate reader (Revvity), and the data were analyzed using GraphPad Prism 9.5 software.

### Degradation Detection Assay‐*Western Blot*

Immunoblotting was carried out as previously described.^[^ ^29^ ^]^ Cells were seeded in 6‐well plates (Yeasen, #84011ES50) at a density of 1 million cells per well. The following day, cells were treated with compounds at the required concentrations, with the DMSO concentration kept below 0.4‰ to prevent toxic effects on the cells. The collected cell pellets were washed three times with PBS (Meilunbio, #MA0015) and resuspended in 120 µL of RIPA lysis buffer (Meilunbio, #MA0151) containing 1% protease inhibitor (Meilunbio, #MB2678‐2). The cells were then sonicated on ice and centrifuged at 12 000 rpm for 15 min at 4 °C to remove cellular debris. Protein concentrations were determined using the BCA Protein Quantification Kit (Meilunbio, #MA0082‐1). The protein samples were normalized, boiled at 99 °C for 10 min, and then subjected to electrophoresis on a 10% SDS‐PAGE gel. The separated protein bands were transferred to nitrocellulose membranes, blocked with TBST containing 5% BSA, and incubated with the indicated primary antibodies. Finally, the blots were imaged using an Amersham Typhoon NIR imager. The following antibodies were used in this experiment: TEAD1 (CST, #12292S, 1:1000, routine assays; Epizyme, #R011985, 1:1000, NCI‐H1299 (TEAD1‐fusion) gradient assay), TEAD3 (Proteintech, #13120‐1‐AP,1:1000), TEAD4 (ABclonal, #A23774,1:1000), CRBN (CST, #71810S, 1:1000), Pan‐TEAD (CST, #13295S, 1:1000), CYR61 (CST, #14479S, 1:1000), PTX3 (ABclonal, #A12669, 1:1000), HiBiT(Promega, #N7200, 1:1000), β‐actin (ABclonal, #AC004,1:2000), IRDye 680RD Goat anti‐Mouse IgG (LI‐COR,# 926–68070,1:5000), IRDye 800CW Goat anti‐Rabbit IgG (LI‐COR,# 926–32211,1:5000).

### HiBiT Assays

HiBiT cells were inoculated into 96‐well flat‐bottomed tissue culture plates at a density of 50 000 cells per well, followed by adding degraders into the wells using the I‐DOT dispenser. After 6 h of incubation, the cells were lysed and assayed for degradation using the Nano‐Glo HiBiT Lysis Assay System (Promega, #N3030). Luminescence signals were read using an Envision plate reader (Revvity) and the data were analyzed using GraphPad Prism 9.5 software.

### Flowcytometry

The TEAD1‐mNeonGreen knock‐in NCI‐H1299 cells were seeded in six‐well plates and treated with the drug at the indicated concentrations. After 24 h, cells were collected and analyzed using an Aurora flow cytometer (Cytek Biosciences). A significant shift in fluorescence intensity was observed if the drug induced a high level of degradation.

### FP‐based TEAD‐YAP Binding Assays

The YBD domains of hTEAD1‐4 were purified as previously described.^[^ ^13^, ^15^ ^]^ In fluorescence polarization (FP) assays, 100 nM TEAD‐YBD protein and 10 nM FAM‐labeled YAP1 peptide were mixed in a total volume of 30 µL assay buffer (20 mM Hepes pH 7.4, 50 mM NaCl, 5% glycerol and 0.01% tween 20) in a 384‐well black plate (Taidu Biotechnology, #GC1238). Compounds were added using an I‐DOT dispenser, starting at 10 µM with a 3‐fold dilution, and the mixture was incubated for 1 h before FP signals were measured using an Envision plate reader (Revvity). Data were analyzed using GraphPad Prism 9.5 software.

### Construction of TEAD HiBiT Cell Lines by CRISPR/Cas9 Editing

The ribonucleoprotein (RNP) complex was prepared by mixing 2 µg of NLS‐Cas9‐EGFP nuclease (Novoprotein, #E379‐01A), 50 pmol of sgRNA, 10 µL of electrotransfer solution, and 50 pmol of designed donor DNA, then incubated for 10 min at room temperature. One million HEK293T cells were resuspended in 10 µL of electrotransfection solution and mixed with 10 µL of the RNP complex. Electroporation was performed using a Celetrix SP100 electroporator at 480 V. After electroporation, the cells were transferred to antibiotic‐free DMEM complete medium for overnight culture. The following day, EGFP‐positive cells were sorted using a Sony MA 900 cell sorter, plated in a 96‐well cell culture plate (Servicebio, #CCP‐96H), and cultured for 4 weeks. After this period, positive clones were screened using the Nano‐Glo HiBiT Lytic Detection system (Promega, #N3030).

### Cell Viability Assays

Cells were seeded at a density of 1000 cells per well in 96‐well flat‐bottomed cell culture plates and cultured overnight at 37 °C with 5% CO_2_ to allow for cell attachment. The following day, different concentrations of compounds were added using an I‐DOT dispenser. After 72 h, 25 µL of CellTiter‐Meiluncell Luminescent Cell Viability Reagent (Meilunbio, #PWL111‐2) was added and incubated for 15 min at room temperature. Luminescence signals were then measured using an Envision plate reader (Revvity). Data were analyzed using GraphPad Prism 9.5 software.^[^ ^30^ ^]^

### Cell Cycle Assay

Cells were seeded in 6‐well plates at a density of 700 000 cells per well. The following day, compounds at different concentrations were added. After 24 h, the cells were processed according to the instructions of the Cell Cycle and Apoptosis Analysis Kit (Beyotime, #C1052). The cell cycle was then analyzed by Aurora flow cytometry (Cytek Biosciences), and the data were processed using FlowJo 10.9.0 and GraphPad Prism 9.5.

### MSTO‐211H CRBN Knock Out Cell Line Construction

HEK293T cells were seeded in 6‐well plates at a density of 400000 cells per well. The Lenti‐CRISPR‐v2‐sgCRBN vector, along with packaging plasmids pMD2.G (Addgene Plasmid #12 259) and psPAX2 (Addgene Plasmid #12 260) in a 3:1:2 ratio, was co‐transfected into HEK293T cells. The viruses were collected at 48 and 72 h, filtered through 0.45 µm filters, and concentrated using the Lenti‐X Concentrator (TaKaRa, #631 231). A total of 100 µL of concentrated virus was added to 1 million MSTO‐211H cells in the presence of 8 µg mL^−1^ of Polybrene (Beyotime, #C0351). After 72 h of transduction, cells were selected with 2 µg mL^−1^ of puromycin, and monoclonal cells were sorted using a Sony MA900 cell sorter. The knockout efficiency was verified by Western blot.

### RT‐qPCR

Cells were seeded in 6‐well plates at a density of 1 million cells per well. The following day, different concentrations of compounds were added, and cells were collected after 24 h. For wash‐out experiments, cells were pre‐treated with compounds at indicated concentrations for 6 h, then cells were washed three times with PBS, and an equal volume of complete medium without compounds was added and incubated for another 18 h.

RNA was isolated and purified from the samples using the EZ‐10 Rotary Column Total RNA Isolation Kit (Sangon Biotech, B615012). After measuring the RNA concentration, 200 ng of purified RNA was used for genomic DNA removal and reverse transcription to cDNA with the HiScript III 1st Strand cDNA Synthesis Kit (+DNA wiper) (Vazyme, R312‐02). The resulting cDNA was then prepared into a reaction mix according to the ChamQ SYBR qPCR Mix (Vazyme, #q711‐02). Real‐time PCR analysis was performed using a QuantStudio 6 Pro real‐time PCR system (Thermo Fisher Scientific). Relative gene expression was calculated using the 2‐∆∆CT method. The primers used in this study were provided in the Supporting Information (Table S1, Supporting Information)

### Proteomics

Cells were seeded in 6‐well plates at 1 × 10⁶ cells well^−1^ and treated with 100 nM KG‐FP‐003 (*n* = 3). After 24 h incubation, cells were harvested, washed with ice‐cold PBS, and stored at −80 °C. Cell pellets were lysed in 4 volumes of lysis buffer (1% SDS, 1% protease inhibitor cocktail) followed by 3 min sonication on ice. After centrifugation (12 000 g, 10 min, 4 °C), supernatants were collected for BCA protein quantification. Proteins were reduced with 5 mM DTT (56 °C, 30 min), alkylated with 11 mM iodoacetamide (RT, dark, 15 min), and diluted with 200 mM TEAB to reduce urea concentration below 2 M. Trypsin digestion (1:50 enzyme‐to‐protein ratio) was performed at 37 °C overnight followed by additional 4 h digestion. Peptides were desalted using Strata‐X SPE columns. For LC‐MS/MS analysis, peptides were separated on a nanoElute UHPLC system (Bruker Daltonics) using a homemade reversed‐phase column (25 cm × 100 µm) with a 20‐min gradient (0–14 min: 6%–24% B; 14–16 min: 24%–35% B; 16–18 min: 35%–80% B; 18–20 min: 80% B) at 500 nL min^−1^, where mobile phase A was 0.1% formic acid/2% acetonitrile and B was 0.1% formic acid/acetonitrile. MS analysis was performed on a timsTOF Pro mass spectrometer in dia‐PASEF mode (ESI voltage: 1.75 kV; scan range: 300–1500 m z^−1^). DIA data were processed using DIA‐NN (v1.8) against the Homo_sapiens_9606_SP_20 231 220.fasta database (20429 entries) with reversed decoys, using trypsin as the cleavage enzyme. Fixed modifications included N‐terminal methionine excision and carbamidomethylation of cysteine, with FDR set at <1%.

### PRISM Assay

The principle of the PRISM assay has been described elsewhere and was conducted as previously outlined by the PRISM team at the Broad Institute.^[^ ^14^, ^31^ ^]^ In PRISM (MTS027) experiments, KG‐FP‐003 was tested across 867 different cancer cell lines. Cells were treated with eight concentration gradients, ranging from 4.6 nM to 10 mM, in triplicate dilutions over a period of five days. Each plate was treated with 20 µM bortezomib as a positive control and DMSO as a negative control. Three replicate experiments were performed for each compound. The resulting data were analyzed using GraphPad PRISM 9.5.

### Pharmacokinetic Evaluation of KG‐FP‐003

All PK experiments were conducted by Medsyin (Shanghai) Co., Ltd. in compliance with the guidelines of the Association for Assessment and Accreditation of Laboratory Animal Care International (AAALAC) under an Institutional Animal Care and Use Committee (IACUC)‐approved protocol (PZWH‐2024052703), using six‐week‐old male ICR mice from Beijing Vital River Laboratory Animal Technology Co., Ltd. housed at 23–25 °C with a 12 h light/dark cycle. Following overnight fasting, three mice received 5 mg kg^−1^ KG‐FP‐003 (in 5% DMSO/95% 20% HP‐β CD) via intraperitoneal injection, with plasma samples collected at 15 min, 30 min, 1, 2, 4, 8, and 24 h post‐dosing. For analysis, 10 µL plasma was protein‐precipitated with 100 µL methanol/acetonitrile (1:1) containing 500 nM tolbutamide (IS), vortexed for 10 min, centrifuged at 4000 rpm for 10 min, and 8 µL supernatant was analyzed by LC‐MS/MS using an AB Sciex 6500 system coupled with Shimadzu LC‐30AD HPLC with Waters CORTECS UPLC T3 column (2.1 × 50 mm, 2.7 µm) at 40 °C, employing a 0.7 mL min^−1^ gradient of 0.1% formic acid in water (A) and acetonitrile (B) (75%→5% A over 0.2–1.5 min). KG‐FP‐003 was quantified via MRM (m/z 525.6/443.3) with tolbutamide (m/z 271.0/74.1) as IS, showing linearity (1–5000 ng mL^−1^) with 2 µL injection volume, and pharmacokinetic parameters were calculated using Phoenix WinNonlin with data expressed as mean ± SD.

### Xenograft Tumor Models‐Pharmacodynamic study

Animal experiments were conducted by Medsyin (Shanghai) Co., Ltd. The experimental protocol was approved by the IACUC of the company (No.: PZWH20240527‐1) and strictly adhered to the guidelines of AAALAC. The specific procedures are as follows: 5 × 10^6^ MSTO‐211H cells were suspended in 100 µL of a mixture of PBS and Matrigel and inoculated subcutaneously into the right axilla of 6‐week‐old BALB/c nude mice. Drug administration was initiated on day 7 after cell inoculation (when the average tumor volume was ≈150 mm^3^). The experiment was divided into two groups: one group served as the control group, and the other group received drug administration via intraperitoneal injection at a dose of 3 mg kg^−1^ (6 mice in each group), once a day for 21 consecutive days. During the experiment, the body weight and tumor volume of the mice were measured twice a week. At the end of the experiment, the mice were sacrificed and tumor tissues were collected. The formula for calculating the tumor growth inhibition rate (TGI) is: TGI = (1 − ΔT/ΔC) × 100%, where ΔT represents the final tumor volume change in the drug‐treated group, and ΔC represents the final tumor volume change in the control group.

### Efficacy Study

Animal experiments were conducted by Medsyin (Shanghai) Co., Ltd. The experimental protocol was approved by the IACUC of the company (No.: PZWH‐2025031003) and strictly adhered to the guidelines of the AAALAC. The specific procedures are as follows: 5 × 10^6^ MSTO‐211H cells or ES2 cells were suspended in 100 µL of a mixture of PBS and Matrigel and inoculated subcutaneously into the right axilla of 6‐week‐old BALB/c nude mice. Drug administration was initiated on day 7 after cell inoculation (when the average tumor volume was ≈150 mm^3^). The experiment was divided into four groups with 6 mice in each group. The specific grouping and administration methods are as follows: control group, KY‐001 group (10 mg kg^−1^, p.o.), KG‐FP‐003 (10 mg kg^−1^ or 30 mg kg^−1^, i.p.). The administration routes included intraperitoneal injection and intragastric administration, once a day for 21 consecutive days. During the experiment, the body weight and tumor volume of the mice were measured twice a week. At the end of the experiment, the mice were sacrificed and tumor tissues were collected. The formula for calculating the tumor growth inhibition rate (TGI) is: TGI = (1 − ΔT/ΔC) × 100%, where ΔT represents the final tumor volume change in the drug‐treated group, and ΔC represents the final tumor volume change in the control group.

### Toxicological Study

A toxicological evaluation was conducted using an MSTO‐211H xenograft mouse model. All animal studies were carried out in accordance with protocols approved by the Institutional Animal Care and Use Committees of Tongji University and the Chinese Academy of Sciences, and in compliance with national, institutional and local regulations. These experiments were conducted in specific‐pathogen‐free (SPF) and Helicobacter‐free facilities at both institutions. After 7 consecutive days of drug administration (10 mg kg^−1^ or 30 mg kg^−1^, i.p., q.d.), whole blood and major organs—including the heart, liver, spleen, lungs, and kidneys—were collected for analysis. Blood samples were anticoagulated with EDTA (1.5 mg mL^−1^), gently mixed, and subjected to standard hematological assessment. Tissue samples were processed for both paraffin‐embedded and frozen section analysis. Paraffin embedding involved fixation, graded dehydration, clearing, embedding, sectioning at 4 µm, and dewaxing to water. Frozen sections were prepared by embedding tissues in OCT compound, sectioning at −20 °C with a thickness of 8 µm, followed by acetone fixation and rinsing with PBS. Hematoxylin and eosin (H&E) staining was performed using the following protocol: hematoxylin staining (3–5 min) → differentiation → bluing → eosin staining (5 min) → graded ethanol dehydration → xylene clearing → mounting with neutral balsam. Microscopic examinations and image acquisition were carried out, and quantitative analyses were performed using specialized image analysis software. Quality control procedures were implemented throughout the experimental workflow—including sample handling, staining protocols, and data processing—to ensure accuracy, reproducibility, and data integrity. *Data analysis was performed using SlideViewer*.

### Molecular Docking

Structure Preparation and Pretest. Protein‐protein docking poses were initially generated using ClusPro 2.0, a user‐friendly free online server.^[^ ^32^ ^]^ The structures of the two ligand subunits and their respective binding pockets were previously determined through crystal structures of their binary complexes. For the docking input, we utilized holo‐form protein structures derived from individual protein‐ligand binary crystals or a ternary crystal. Subsequently, the top two poses with “balanced” scores from ClusPro 2.0 were chosen for further refinement in the molecular docking process. The docking simulation involving the KG‐FP‐003 and CRBN‐TEAD1 aligned complex was carried out using Surflex‐Dock Geom, a tool available in Sybyl‐X version 2.1.1 (Trepos, LLC).

### Transcriptomics Analysis

A172 cells were seeded into 6‐well plates at a density of 1.5 × 10⁶ cells per well and treated with DMSO (control), 30 nM KG‐FP‐003, or 30 nM IAG933 for 24 h (*n* = 3 per group). Following treatment, cells were harvested, washed three times with pre‐chilled PBS, and stored at −80 °C. Total RNA was extracted using TRIzol reagent according to the manufacturer's instructions. mRNA was enriched using oligo(dT) magnetic beads, followed by fragmentation in First Strand Synthesis Reaction Buffer (5×) with divalent cations under elevated temperature.

RNA‐seq libraries were constructed using the ABclonal Fast RNAseq Lib Prep Kit V2 (#RK20306). After quality control, libraries were pooled according to their effective concentrations and sequenced on an Illumina platform using sequencing‐by‐synthesis (SBS) technology. Base calling was performed through real‐time fluorescence capture of polymerase‐driven extension reactions using fluorescently labeled dNTPs and adapter‐anchored primers. Gene‐level read counts were quantified using featureCounts (v2.0.6) and normalized to FPKM values by incorporating gene length. Differential expression analysis was conducted using the DESeq2 package (v1.42.0) for samples with biological replicates, based on the negative binomial distribution. P‐values were adjusted using the Benjamini–Hochberg method, with significance thresholds set at adjusted p ≤ 0.05 and |log_2_(fold change)| ≥ 1. For samples without replicates, edgeR (v4.0.16) was used, with scaling normalization applied to correct for sequencing depth differences. Functional enrichment analysis, including Gene Ontology (GO) and KEGG pathway analysis, was performed using clusterProfiler (v4.8.1), with correction for gene length bias. A significance threshold of adjusted p < 0.05 was applied. In addition, Gene Set Enrichment Analysis (GSEA) was performed to assess coordinated expression changes in predefined gene sets (GO and KEGG), with genes ranked by the magnitude of differential expression. All transcriptomic experiments were performed by Novogene Co., Ltd.

### Co‐Immunoprecipitation

HEK293T cells were seeded in 6‐well plates at a density of 1.5 × 10^6^ cells per well and cultured overnight in DMEM supplemented with 1% FBS under standard conditions (37 °C, 5% CO_2_). When cells reached 60%–70% confluency, plasmid transfection was performed. Flag‐TEAD and pCMV‐HA‐Ub plasmids were mixed at a 1:1 ratio and diluted in Opti‐MEM to a final volume of 250 µL (solution A). In parallel, Lipofectamine 2000 was diluted in Opti‐MEM at a ratio of 3 µL reagent per 1 µg plasmid DNA, adjusted to a final volume of 250 µL (solution B). Solutions A and B were combined, incubated briefly, and added to the cells. After 24 h of transfection, cells were treated with either DMSO (control) or KG‐FP‐003 (100 nM). To prevent degradation of ubiquitinated proteins, bortezomib (0.4 µM) was added 2 h prior to compound treatment. Subsequently, cells were lysed and processed for immunoprecipitation following the manufacturer's protocol (Beyotime, #P2181S). The precipitated complexes were analyzed by Western blotting to detect protein expression and interactions.^[^ ^24^ ^]^

### Statistical Analysis

All statistical analyses were performed using GraphPad Prism 9.5. Data from cell viability assays, RT‐qPCR, and tumor growth curves were analyzed using either one‐way ANOVA or unpaired Student's t‐test, as appropriate. Statistical significance was determined as follows: ns, not significant; *, *p* < 0.05; **, *p* < 0.01; ***, *p* < 0.001; ****, *p* < 0.0001. All results were presented as the mean ± standard deviation (SD) from at least three independent biological replicates.

## Conflict of Interest

Y. Shi, Y. Zhang, J. Chen, Y. Wang, and K. Zhao are employees of Kygent Therapeutics (Shanghai) and may hold stock shares in the company. Y. Shi, Y. Zhang, and J. Chen are co‐inventors of patent CN116217554B, and a provisional patent related to this publication.

## Supporting information



Supporting Information

Supplemental Data

## Data Availability

The data that support the findings of this study are available on request from the corresponding author. The data are not publicly available due to privacy or ethical restrictions.
