# Supplementary material for: Discovery of a Potent and Selective TEAD Degrader with Durable Degradation Activity
Source: Adv Sci (Weinh). 2025 Oct 5;12(48):e03277. doi: 10.1002/advs.202503277 (PMC12752627; doi:10.1002/advs.202503277)
Supplement: Supplementary file 1 — Supporting Information [file ADVS-12-e03277-s002.docx]

**Discovery of a Potent and Selective TEAD Degrader with Durable Degradation Activity**

Linhui Cao^a,b,^*, Jing Yang^c,^*, Yuhang Liu^a,b^, Xiaotong Chen^c^, Yufang Shi^d^, Yunshuo Zhao^c^, Yong Zhang^d^, Jian Chen^d^, Bowen Li^c^, Wuqiang Wen^e^, Lu Chen^b^, Bo Peng^b^, Lu Huang^b,f^, Yanli Sun^b^, Lixin Zhou^b,c^, [Matthew G Rees](https://pubmed.ncbi.nlm.nih.gov/?term=Rees+MG&cauthor_id=36946421)^g^ , [Melissa M Ronan](https://pubmed.ncbi.nlm.nih.gov/?term=Ronan+MM&cauthor_id=36946421)^g^ , [Jennifer A Roth](https://pubmed.ncbi.nlm.nih.gov/?term=Roth+JA&cauthor_id=36946421)^g^ , Zhixiang Guo^f^, Jing Xing^b^, Guangya Zhu^b^, Yazhou Wang^d^, Baishan Jiang^e^, Jing Lu^a^, Kehao Zhao^a,d, #^, Wenchao Lu^b, #^

^a^School of Pharmacy, Key Laboratory of Molecular Pharmacology and Drug Evaluation (Yantai University), Ministry of Education, China; Collaborative Innovation Center of Advanced Drug Delivery System and Biotech Drugs in Universities of Shandong, Yantai University, Yantai 264005, China

^b^Lingang Laboratory, Shanghai 200031, China

^c^Department of Hematology, Tongji Hospital, Frontier Science Center for Stem Cell Research, Shanghai Key Laboratory of Signaling and Disease Research, School of Life Sciences and Technology, Tongji University, Shanghai 200092, China

^d^Kygent Therapeutics, Shanghai 201203, China

^e^Department of Radiation and Medical Oncology, Medical Research Institute, Frontier Science Center of Immunology and Metabolism, Zhongnan Hospital of Wuhan University, School of Pharmaceutical Sciences, Wuhan University, Wuhan 430071, China

^f^Department of Cardiovascular Surgery, The First Affiliated Hospital of Anhui Medical University, 218 Jixi Road, Hefei 230022, China.

^g^Broad Institute of MIT and Harvard, Cambridge, Massachusetts 02142, United States.

*These authors contributed equally

^#^Corresponding author

E-mail: [luwenchao@lglab.ac.cn](mailto:luwenchao@lglab.ac.cn) (Lu W.)

E-mail: [kehaozhao@gmail.com](mailto:kehaozhao@gmail.com) (Zhao K.)

**Supplementary Figures**

**Chemistry**

**PROTAC Synthesis：**

**Reagents and conditions:**（a）DCE，NaHB(Ac)_3，_80°C，16h，27% yield; (b) 1) NH_3_/MeOH, 2) NaBH(OAc)_3_, DCE, 80 °C, 2 h, 45%yield; (c) LiOH, THF, H_2_O, 0~r.t, 2 h, 94.5% yield; (d) HATU, DIEA, DMF, r.t, 2 h, 68.8% yield; (e) TCFH, NMI, DMF, r.t, 16 h , 49.2% yield; (f) HCl, MeOH, r.t, 1 h, 65.6% yield.

***Synthesis of compound A_1_.*** Dissolve compound A (390 mg, 0.64 mmol) and trans-4-aminocyclohexanecarboxylic acid methyl ester (200 mg, 1.28 mmol) in 1,2-dichloroethane (8 mL), add acetic acid (19 mg, 0.32 mmol), react at 80 °C for 15 hours, and add sodium borohydride acetate (270 mg, 1.28 mmol). LCMS shows complete reaction of the raw materials. Dilute the reaction solution with water, extract with dichloromethane (50 mL × 3), and wash the combined organic phases with saturated saline solution (20 mL × 3). Dry the organic phases with anhydrous sodium sulfate, filter, and concentrate the filtrate under reduced pressure. Then, purify in reverse phase using C18 (ACN: H2O=60%) to obtain the yellow solid compound A1(130 mg, 27.0%). Compound A1: LC-MS m/z (ESI):753.4[M+H]^+^。

***Synthesis of compound A_2_.*** Dissolve compound A1 (130 mg, 0.17 mmol) in tetrahydrofuran (2 mL), and add 8.5 mg of lithium hydroxide, 0.35 mmol) was dissolved in water (1 mL) and added to the above system at 0 °C. The reaction was carried out at room temperature for 2 hours, and LCMS showed complete reaction of the raw materials. Adjust the reaction solution to pH 3 with hydrochloric acid (2M), extract with dichloromethane (50 mL × 3), wash the combined organic phases with saturated saline solution (20 mL × 3), dry the organic phase with anhydrous sodium sulfate, filter, and concentrate the filtrate under reduced pressure to obtain the crude product Compound A_2_ (120 mg, crude) as a yellow solid compound A_2_: LC-MS m/z (ESI):739.3[M+H]^+^.

***Synthesis of compound B.*** Dissolve compound A (290 mg, 0.49 mmol) in 1,2-dichloroethane (3.0 mL) under nitrogen protection. Add ammonia methanol solution (0.7 mL, 7 M) and acetic acid (2.94 mg, 0.0049 mmol) in sequence. Heat up to 80 °C and stir for 2 hours. Then add sodium borohydride acetate (154 mg, 2.45 mmol) and stir at 80 °C for 30 minutes. LCMS shows complete reaction of the raw materials. Cool down to 25 °C and pour the reaction solution into saturated sodium bicarbonate (20 mL) for quenching. Extract with ethyl acetate (20 mL × 3). Combine the organic phases and wash with saturated saline solution (20 mL). Dry the organic phase with anhydrous sodium sulfate, filter, and concentrate the filtrate under reduced pressure. Purification by silica gel column chromatography (EA: PE=1:10~1:2) resulted in white solid compound B (134 mg, Y: 45.3%). LC-MS m/z (ESI): 612.2 [M-H]^-^.

***Synthesis of compound KG-FP-008a*.** Dissolve compound B (100 mg, 0.16 mmol) in N, N-dimethylformamide (2.0 mL), cool to 0 °C, and sequentially add 5-((2-(2,6-dioxopiperidin-3-yl)-1,3-dioxoisoindolin-5-yl)amino)-5-oxopentanoic acid (80.0 mg, 0.14 mmol), N,N,N’,N’– tetramethylchloroformamidine hexafluorophosphate (140 mg, 0.85 mmol), and N-methylimidazole (340 mg, 4.09 mmol). React at room temperature under nitrogen protection for 16 hours. TLC (DCM: MeOH=10:1) showed complete reaction of the raw materials. Add saturated sodium bicarbonate aqueous solution (50 mL) to the reaction solution, extract with dichloromethane (50 mL × 3), wash the combined organic phases with saturated saline solution (50 mL × 2), dry the organic phase with anhydrous sodium sulfate, filter, concentrate the filtrate under reduced pressure, and purify by silica gel column chromatography (EA: PE=1:10~1:1) to obtain white solid compound KG-FP-008a (80 mg, Y: 49.2%). LC-MS m/z (ESI): 996.6 [M+H]^+^.

***Synthesis of compound KG-FP-008.*** Dissolve compound KG-FP-008a (70 mg, 0.070 mmol) in hydrochloric acid methanol solution (1 mL, 4.0 M) and react at room temperature for 30 minutes. LCMS shows complete reaction of the raw material. Concentrate the reaction solution under reduced pressure to a yellow solid compound (50 mg, crush), and efficiently prepare the liquid phase (column model: YMC-Actus Triart C18, 150*20mm，5um；Eluent: 0.05% NH_3_H_2_O-10 mmol/L NH_4_HCO_3_-ACN; Gradient: 34-54 MeCN in H_2_O; Flow rate: 20 ml/min; Running time: 18 min; Purification with peak time Rt=12 min resulted in white solid compound KG-FP-008 (15 mg, Y: 24.2%). ^1^H NMR (400 MHz, DMSO-*d*_6_) δ 11.09 (s, 1H), 10.50 (s, 1H), 8.35-8.25 (m, 2H), 7.92-7.79 (m, 3H), 7.40-7.25 (m, 6H), 7.00 (d, *J* = 9.6 Hz, 1H), 5.12 (dd, *J* = 12.8, 5.9 Hz, 2H), 4.95 (t, *J* = 6.0 Hz, 1H), 3.69 (q, *J* = 7.1 Hz, 1H), 3.52 (dd, *J* = 14.2, 4.8 Hz, 1H), 3.43 (d, *J* = 19.2 Hz, 1H), 2.98-2.84 (m, 3H), 2.71 (dd, *J* = 16.1, 9.8 Hz, 1H), 2.64 (d, *J* = 4.4 Hz, 3H), 2.44-2.37 (m, 1H), 2.31 (t, *J* = 7.0 Hz, 2H), 2.11-1.99 (m, 3H), 1.48-1.24 (m, 5H), 1.10 (d, *J* = 6.9 Hz, 3H). LC-MS m/z (ESI): 882.3[M+H]^+^.

***Synthesis of compound KG-FP-003a.*** Dissolve compounds A_2_ (120 mg, 0.16 mmol) and 2-(2,6-dioxopiperidin-3-yl)-5-(2-oxo-2-(4-(piperidin-4-ylmethyl)piperidin-1-yl)ethoxy)isoindoline-1,3-dione (79 mg, 0.18 mmol) in N, N-dimethylformamide (2 mL), add 2- (7-azobenzotriazole) - N, N, N',N' - tetramethylurea hexafluorophosphate (93 mg, 0.24 mmol) and N, N-diisopropylethylamine (63 mg, 0.49 mmol), and react at room temperature for 2 hours. LC-MS shows complete reaction of the raw materials. The reaction solution was purified in reverse phase using C18 (ACN: H_2_O=32%) to obtain a pale yellow solid compound KG-FP-009a (130 mg, 68.8%). KG-FP-003a: LC-MS m/z (ESI):1164.4[M+H]^+^.

***Synthesis of compound KG-FP-009a.*** Dissolve compound A_2_ (100 mg, 0.13 mmol) and 2-(2,6-dioxopiperidin-3-yl)-5-(2-oxo-2-(4-(piperidin-4-ylmethyl)piperidin-1-yl)ethoxy)isoindoline-1,3-dione (80 mg, 0.16 mmol) in dichloromethane (4 mL), add 2- (7-azobenzotriazole) - N, N, N', N' - tetramethylurea hexafluorophosphate (102 mg, 0.27 mmol) and N, N-diisopropylethylamine (69.9 mg, 0.54 mmol), and react at 25 °C for 2 hours. TLC shows complete reaction of the raw materials. Dilute the reaction solution with water, extract with ethyl acetate (50 mL × 3), wash the combined organic phases with saturated saline (20 mL × 3), dry the organic phases with anhydrous sodium sulfate, filter, and concentrate the filtrate under reduced pressure to obtain light yellow solid compound KG-FP-009a (70 mg, 48%).

***Synthesis of compound KG-FP-010a****.* Dissolve compound 5-amino-N-(3-(2,4-dioxotetrahydro-pyrimidin-1(2H)-yl)-4-methylphenyl)pentanamide (80 mg, 0.25 mmol) and A_2_ (186 mg, 0.25 mmol) in dry dichloromethane (2 mL), and add 2- (7-azabenzotriazole) – N, N,N', N' - tetramethylurea hexafluorophosphate (143 mg, 0.38 mmol) and triethylamine (203 mg, 2.0 mmol) were reacted at 25 °C for 2 hours, and LCMS showed complete reaction of the raw materials. Add water (15 mL) to the reaction solution, extract with ethyl acetate (15 mL × 3), and wash the combined organic phases with saturated saline solution (15 mL × 3). Dry the organic phase with anhydrous sodium sulfate, filter, and concentrate the filtrate under reduced pressure. Purify with C18 reverse phase (CH_3_CN: H_2_O=2:1) to obtain white solid compounds KG-FP-010a (80 mg, Y: 30%). LC-MS m/z (ESI): 1039.7[M+H]^+^.

***General Procedure for Synthesis of Compounds KG-FP-003、KG-FP-009 and KG-FP-010.*** Dissolve compound *KG-FP-003a* or *KG-FP-009*a or *KG-FP-011a* (0.86 mmol) in methanol (1 mL), add hydrochloric acid methanol solution (1 mL, 4 M), and react at 25 °C for 1 hour. LCMS shows complete reaction of raw materials. Add dimethyl sulfoxide (1 mL) to the reaction solution and concentrate under reduced pressure to efficiently prepare the liquid phase (column model: YMC-Actus Triart C18，150*20 mm，5 um； Eluent: 0.05% NH3H2O-10mmoL/L NH_4_HCO_3_-ACN=1:1; Gradient: 37%-57%; Flow rate: 20 mL/min; Running time: 10 minutes. Obtain solid compounds KG-FP-003, KG-FP-009 and KG-FP-011 was purified with a peak time of 9.0 min.

***Synthesis of compound KG-FP-003.*** General procedure f (59.2 mg, white solid, Y: 65.6%). Structural identification data: ^1^H NMR (400 MHz, DMSO-*d*_6_) δ 10.27 (s, 1H), 8.23 (d, J = 4.4 Hz, 1H), 7.39-7.25 (m, 6H), 7.17-7.12 (m, 1H), 7.06-7.01 (m, 1H), 6.82-6.74 (m, 2H), 5.18-5.13 (m, 1H), 4.97-4.92 (m, 1H), 4.80-4.72 (m, 2H), 4.36-4.28 (m, 2H), 3.86-3.66 (m, 3H), 3.51-3.44 (m, 2H), 2.93-2.79 (m, 5H), 2.58-2.51 (m, 7H), 2.44-2.39 (m, 3H), 2.14-2.09 (m, 1H), 2.13 (s, 3H), 1.83-1.56 (m, 10H), 1.30-0.79 (m, 14H). LC-MS m/z (ESI):1049.6 [M+H]^+^。

***Synthesis of compound KG-FP-009*.** General procedure f (20mg, white solid, Y: 27%). Structural identification data: ^1^H NMR (400 MHz, DMSO-*d*_6_) δ 11.10 (s, 1H), 8.25 (s, 1H), 7.83 (d, *J* = 8.2 Hz, 1H), 7.40-7.37 (m, 5H), 7.35-7.30 (m, 3H), 7.06 (d, *J* = 9.3 Hz, 1H), 5.14-5.05 (m, 4H), 4.95 (t, *J* = 6.0 Hz, 1H), 4.39-4.25 (m, 2H), 3.86-3.76 (m, 2H), 3.53-3.43 (m, 1H), 3.12-2.67 (m, 8H), 2.64 (d, *J* = 4.4 Hz, 3H), 2.62-2.54 (m, 3H), 2.44-2.33 (m, 3H), 2.10-1.99 (m, 2H), 1.89-1.75 (m, 2H), 1.71-1.50 (m, 8H), 1.32-1.20 (m, 4H), 1.14-1.09 (m, 6H), 1.00-0.77 (m, 4H). LC-MS m/z (ESI):1103.5 [M+H]^+^.

***Synthesis of compound KG-FP-010*.** General procedure f (21.32mg, white solid, Y: 34%, formate) . Structural identification data: ^1^H NMR (400 MHz, DMSO-*d_6_*): δ 10.32 (s, 1H), 9.87 (s, 1H), 8.31-8.18 (m, 2H), 7.70-7.61 (m, 1H), 7.58-7.50 (m, 1H), 7.43-7.30 (m, 5H), 7.31-7.22 (m, 2H), 7.19 (d, *J*=8.4 Hz, 1H), 7.00 (d, *J*=9.6 Hz, 1H), 5.11 (s, 1H), 4.96 (d, *J*=5.6 Hz, 1H), 3.78-3.67 (m, 1H), 3.52-3.45 (m, 4H), 3.06-2.90 (m, 4H), 2.79-2.68 (m, 4H), 2.63 (d, *J*=4.8 Hz, 3H), 2.42-2.18 (m, 3H), 2.11 (s, 3H), 2.00-1.89 (m, 1H), 1.87-1.70 (m, 2H), 1.69-1.49 (m, 4H), 1.46-1.20 (m, 4H), 1.10 (d, *J*=7.2 Hz, 3H), 0.95-0.78 (m, 2H)。LC-MS m/z (ESI): 925.6[M+H]^+^.

**
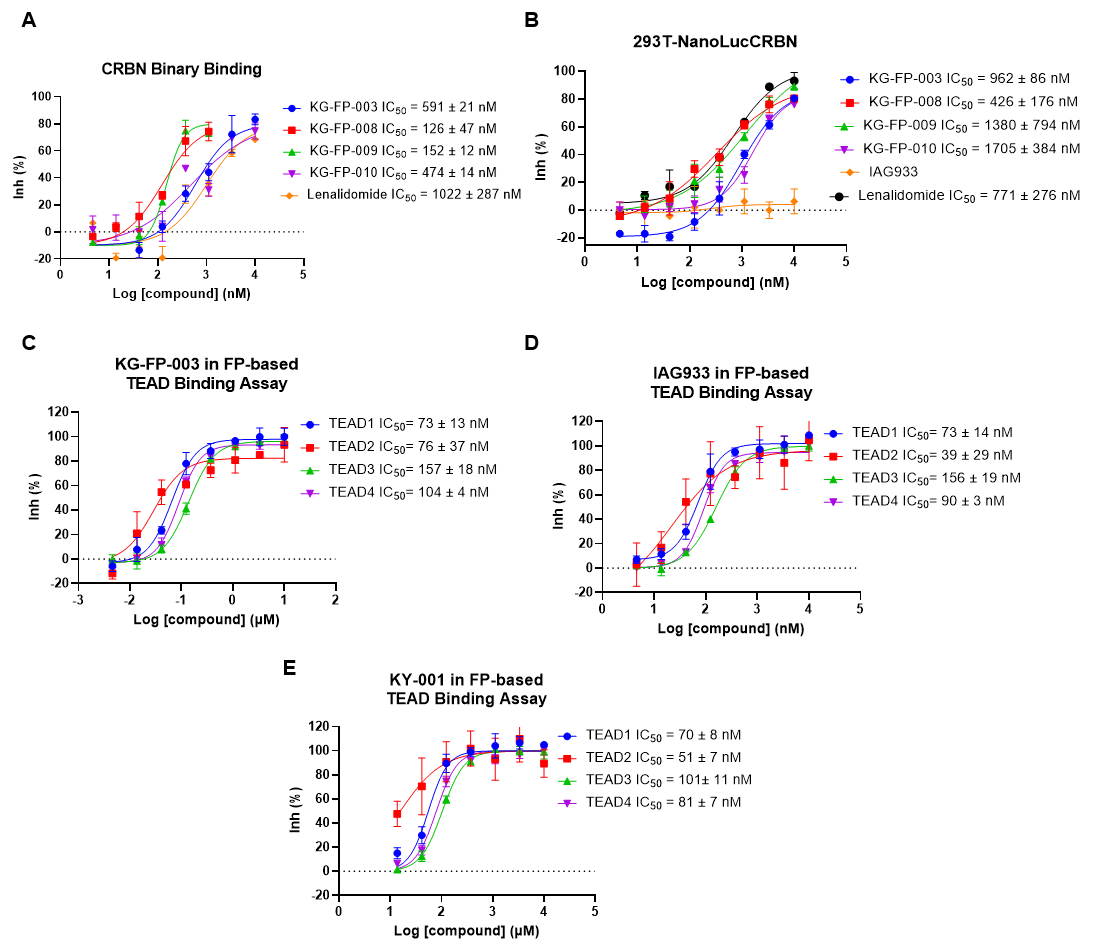
 Biology**

**Fig. S1 Binary binding for KG-FP series and TEAD/YAP warheads. (A)** Biochemical binding affinity of the KG-FP series to recombinant CRBN-DDB1 was measured using an HTRF assay, with lenalidomide serving as the positive control. Data represent three independent experiments. **(B)** Intracellular binding of the KG-FP series to CRBN was evaluated in HEK293T cells overexpressing NanoLuc-CRBN, using lenalidomide as the control. Data represent three independent experiments. **(C-E)** Binding affinity of KG-FP-003 to TEAD-YBD proteins was assessed, with IAG933 and KY-001 as reference controls. Data represent three independent experiments.


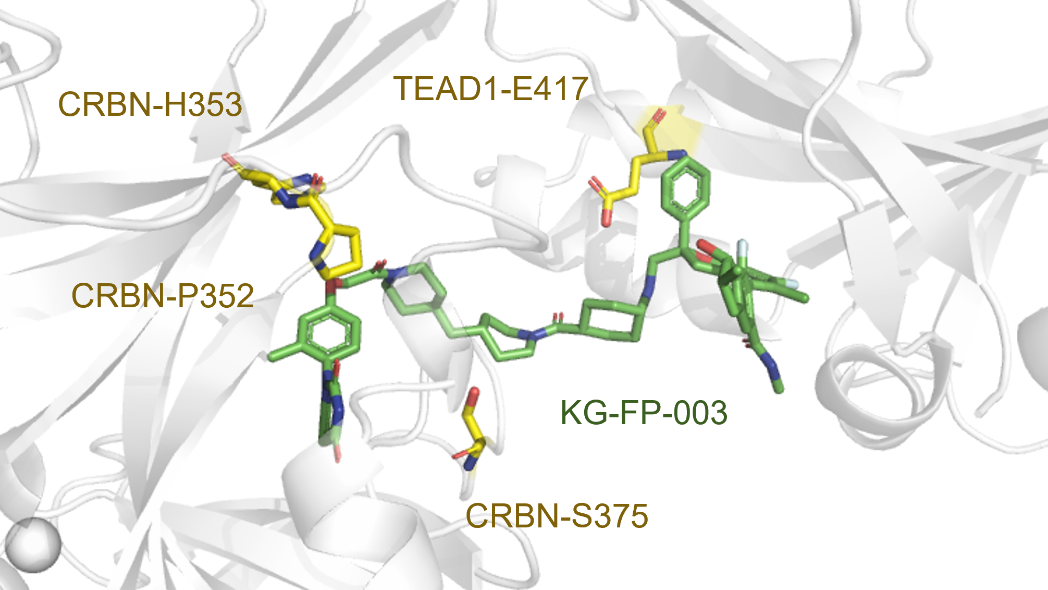


**Fig. S2** Prediction and experimental validation of ternary complex formation for TEAD1-YBD/KG-FP-003/CRBN in NanoBiT assays. Data are representative of three independent experiments.


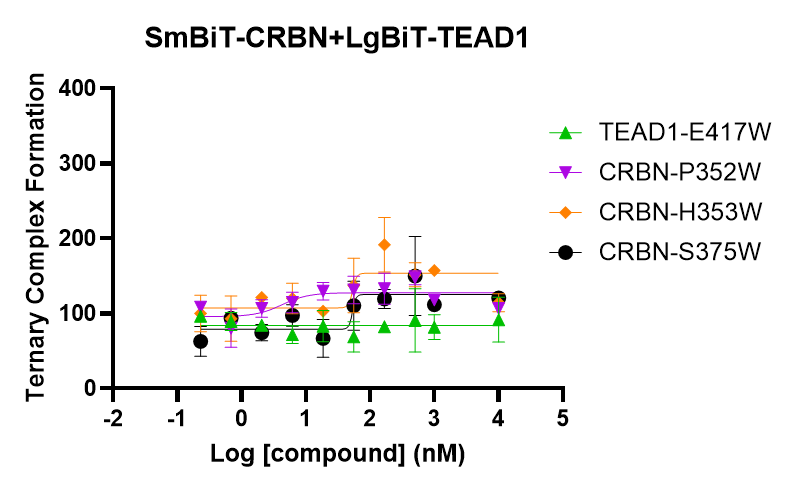


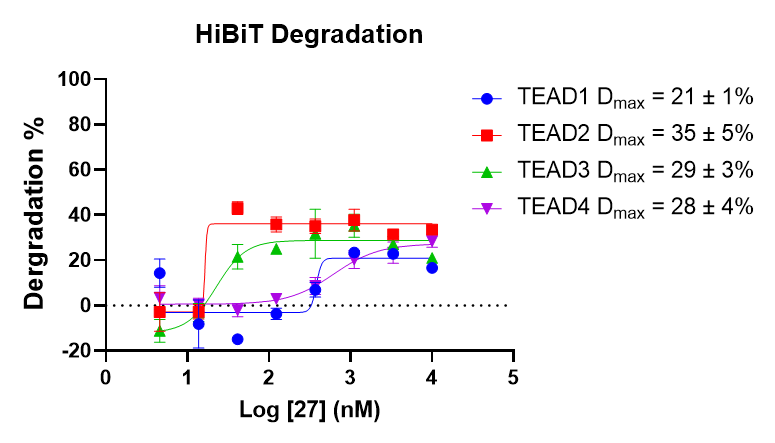


**Fig. S3** Evaluation of the degradation efficacy of LBP inhibitor-based PROTAC 27 on HiBiT-tagged TEAD proteins in HEK293T cells following 6 hours of treatment. Data are representative of three independent experiments.


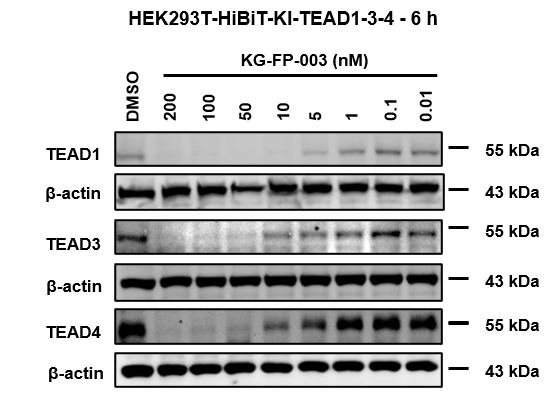


**Fig. S4** Immunoblot analysis of concentration-dependent degradation of TEAD1, TEAD3, and TEAD4 mediated by KG-FP-003 in HEK293T-HiBiT-KI-TEAD1/3/4 cells at 6 hours.


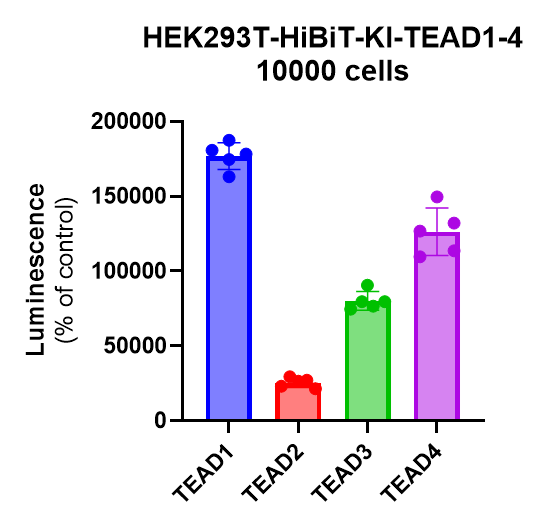


**Fig. S5** Differential signal intensities from equal numbers (10,000 cells) of HEK293T-HiBiT knock-in cells expressing TEAD1, TEAD2, TEAD3, or TEAD4 confirm variations in endogenous TEAD expression levels.


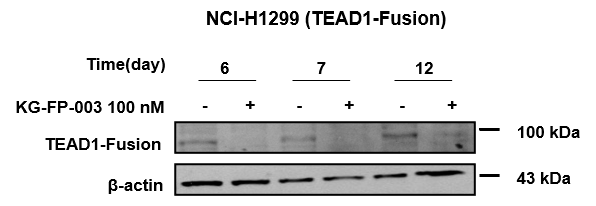


**Fig. S6** Immunoblot analysis of TEAD1-fusion NCI-H1299 cells treated with 100 nM KG-FP-003 for 6, 7, and 12 days, respectively.


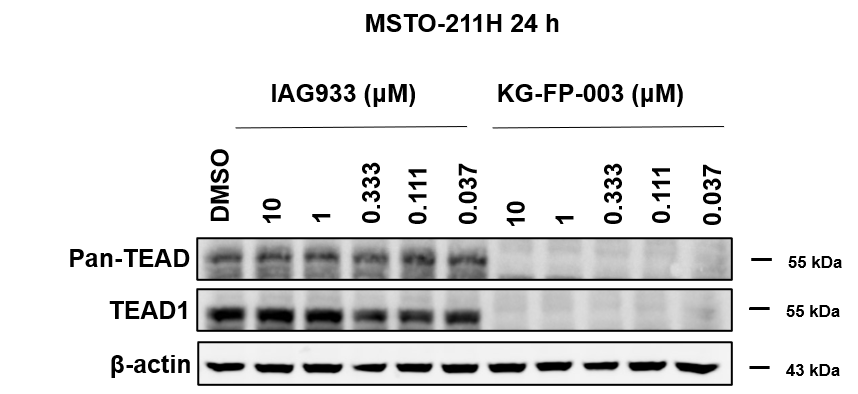


**Fig. S7** Western blot analysis of MSTO-211H cells treated with varying concentrations of KG-FP-003 and IAG933 for 24 hours.


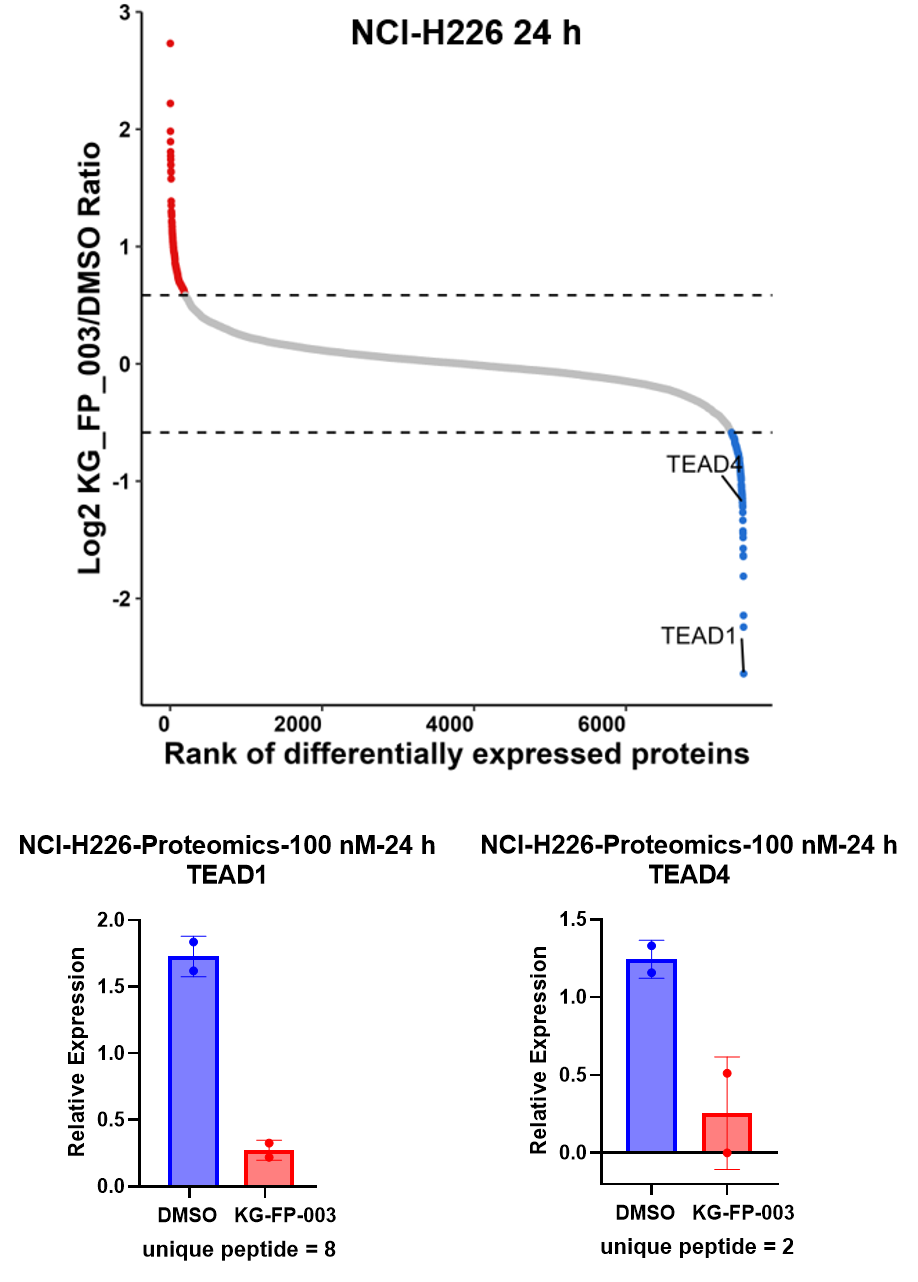


**Fig. S8** DIA-based proteomic analysis of NCI-H226 cells following 24-hour treatment with KG-FP-003: Snake plot visualization and regulation of TEAD1 and TEAD4 expression.


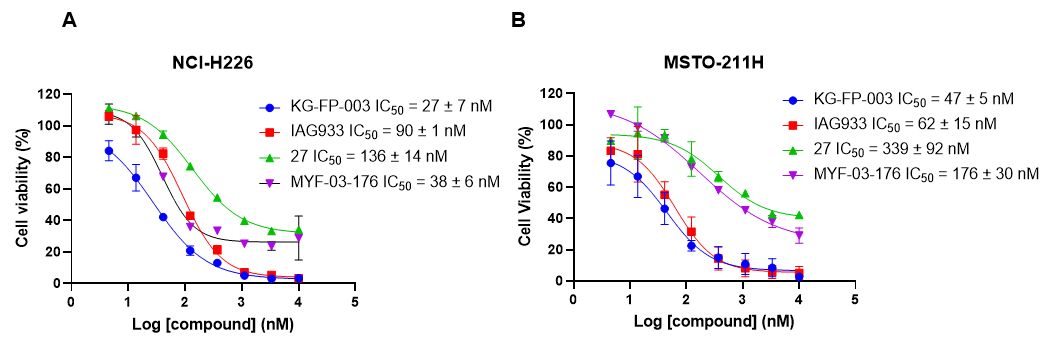


**Fig. S9** CTG cell viability assay of NCI-H226 and MSTO-211H cells treated with indicated compounds at various concentrations for 72 hours. Data represent biological triplicates and are representative of three independent experiments. Data are representative of three independent experiments.

**
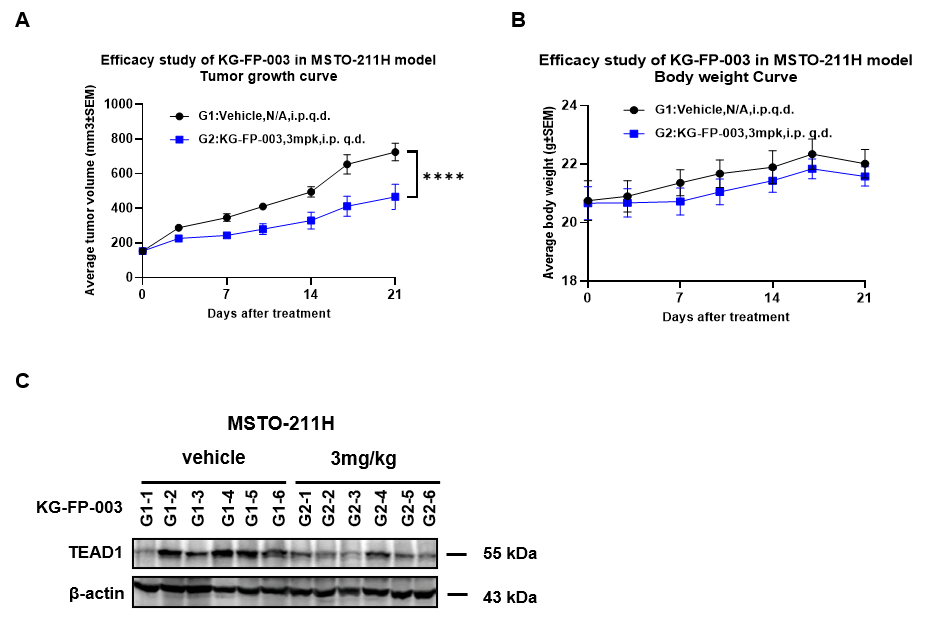
**

**Fig. S10 Pharmacodynamic effects and preliminary efficacy of KG-FP-003 in the MSTO-211H xenograft model. (A)** Tumor growth curves of BALB/c nude mice bearing MSTO-211H xenografts treated with vehicle (DMSO) or KG-FP-003 (i.p., q.d. for 21 days; n = 6). **(B)** Body weight monitoring throughout the treatment course. **(C)** End-point immunoblot analysis of tumor lysates showing TEAD1 degradation following *in vivo* administration of KG-FP-003. Tumor samples were collected 1 hour after the last dose on day 21.


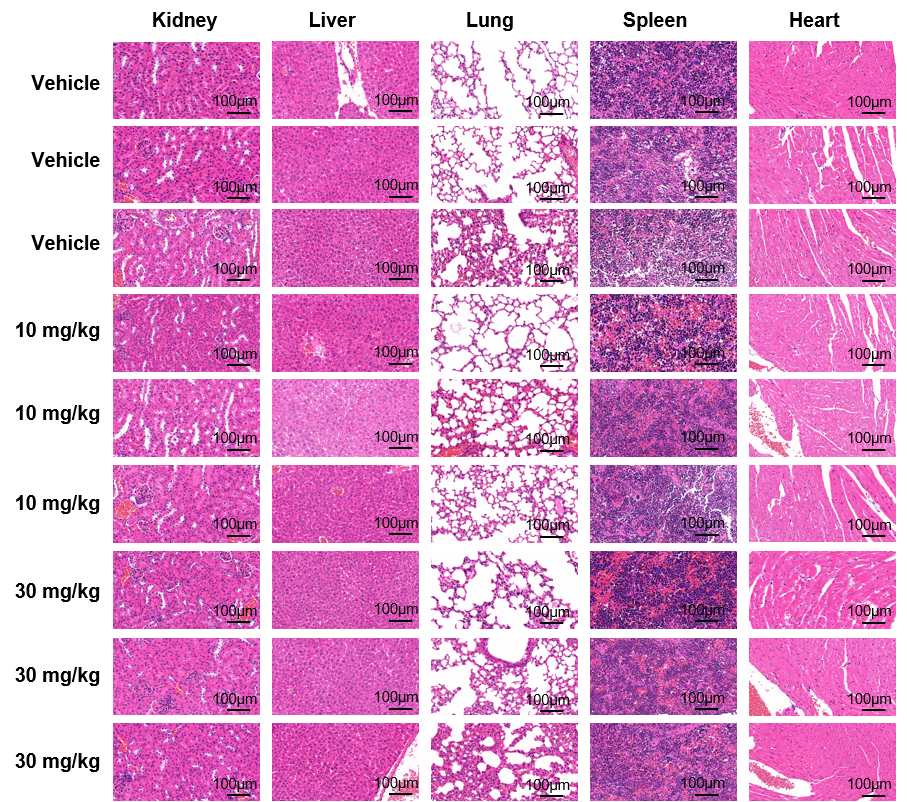


**Fig. S11** Histopathological analysis of representative tissue sections following 7-day *in vivo* administration of KG-FP-003 in mice (i.p., q.d.) for 7 consecutive days, as assessed by hematoxylin and eosin (H&E) staining. Scale bar: 100μm.


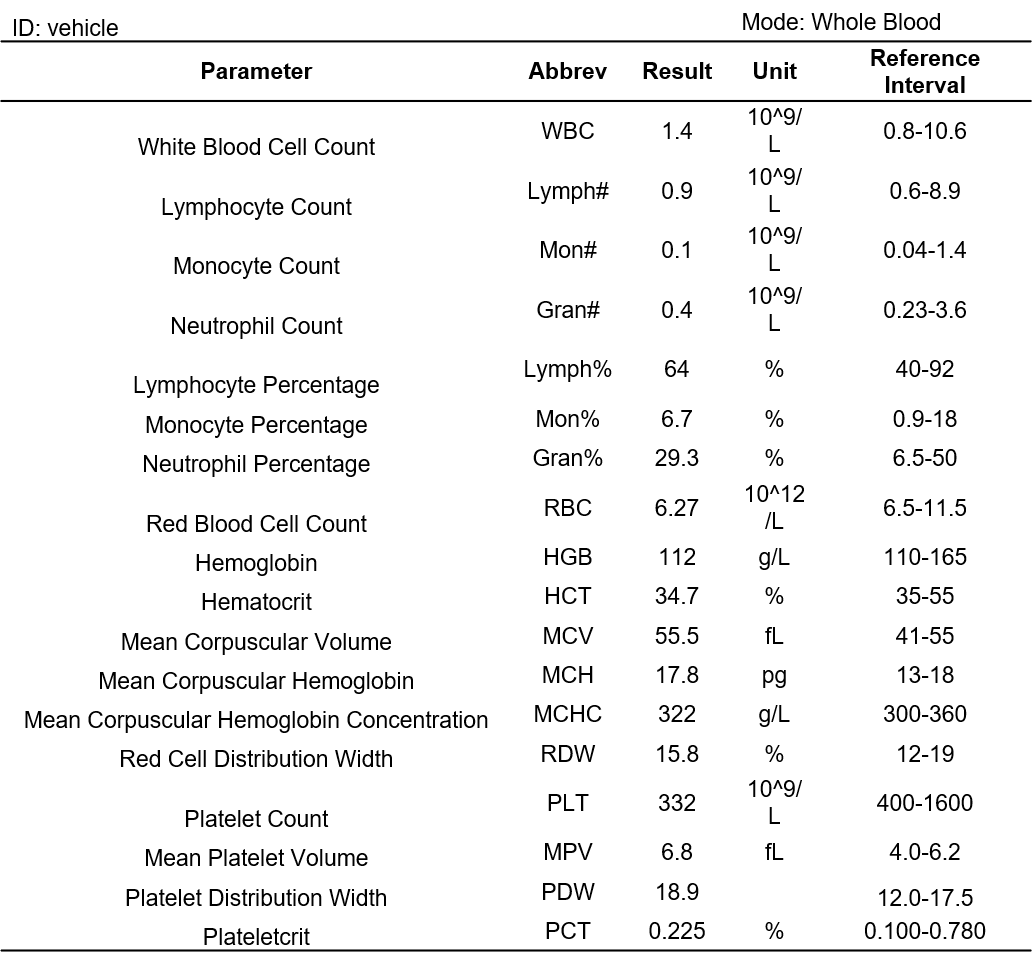
**Fig. S12** Hematological analysis of representative mice treated with vehicle control (i.p., q.d.) for 7 consecutive days.

**Fig. S13** Hematological analysis of representative mice treated with KG-FP-003 (10 mg/kg, i.p., q.d.) for 7 consecutive days.


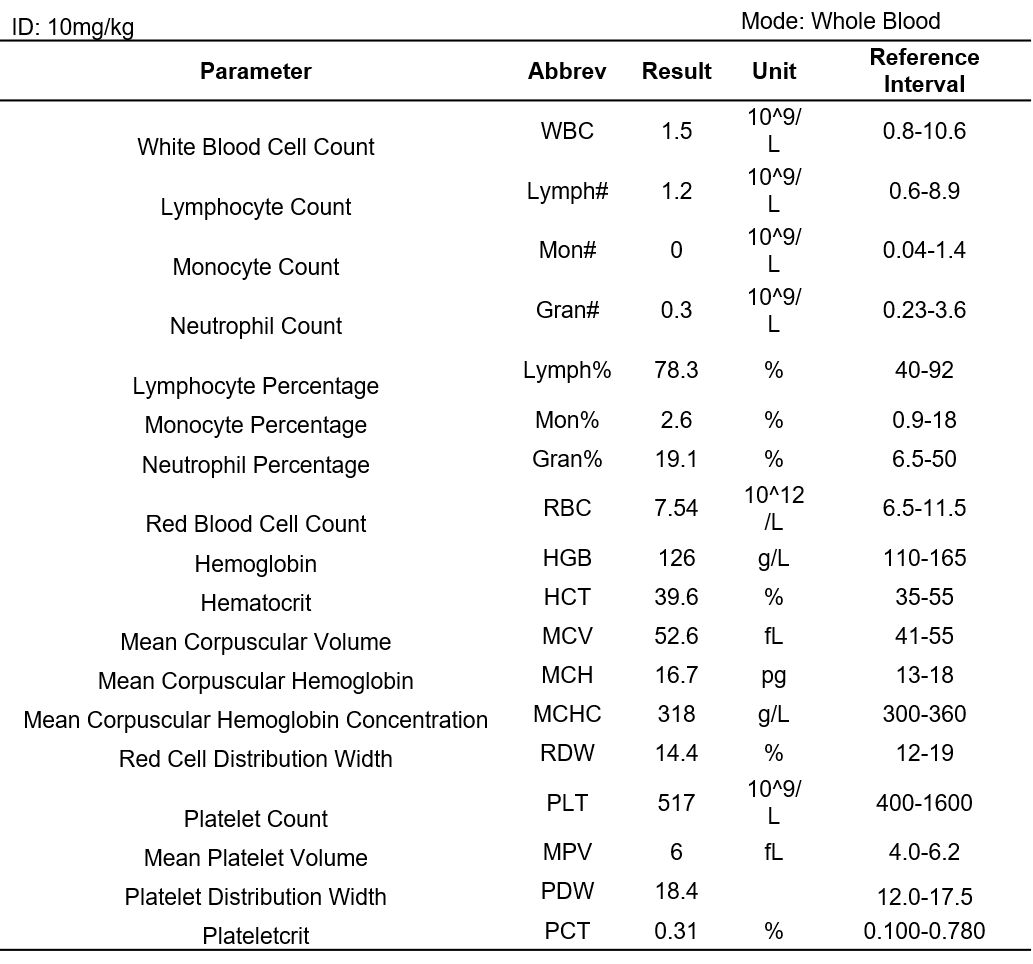


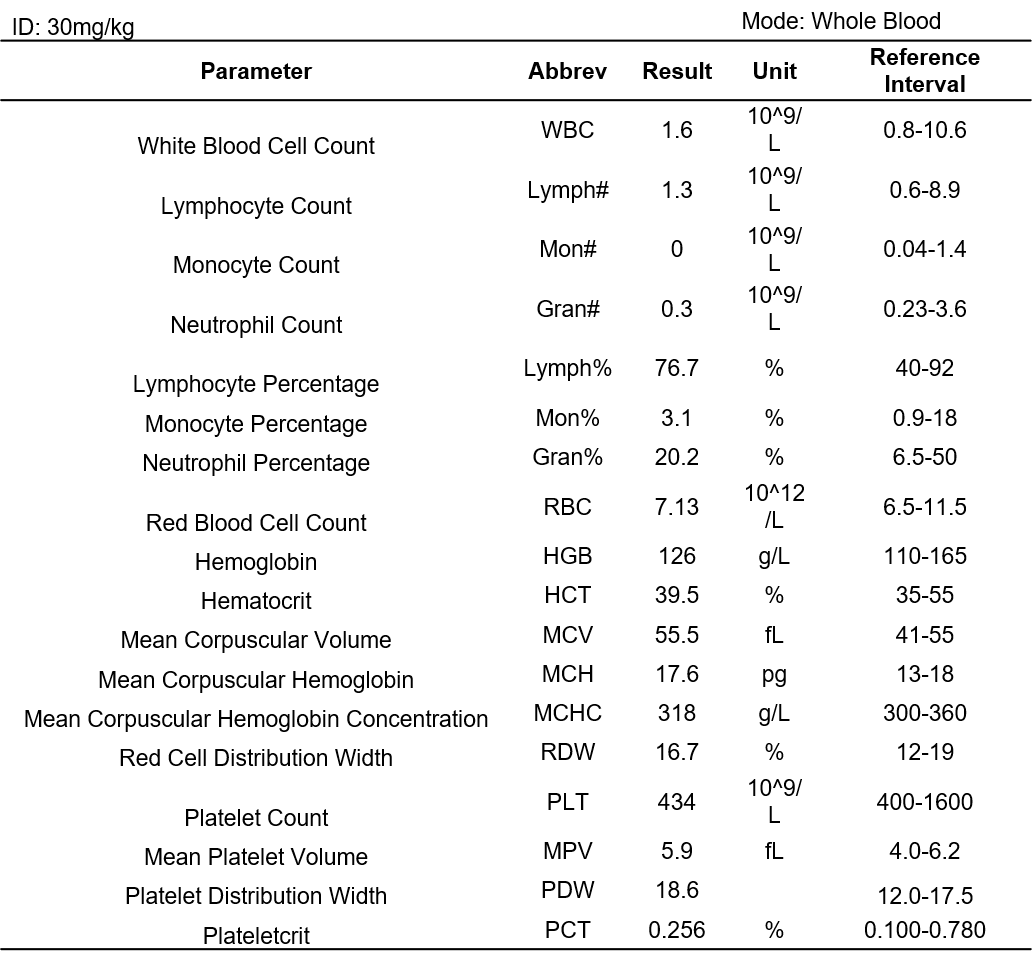


**Fig. S14** Hematological analysis of representative mice treated with KG-FP-003 (30 mg/kg, i.p., q.d.) for 7 consecutive days.


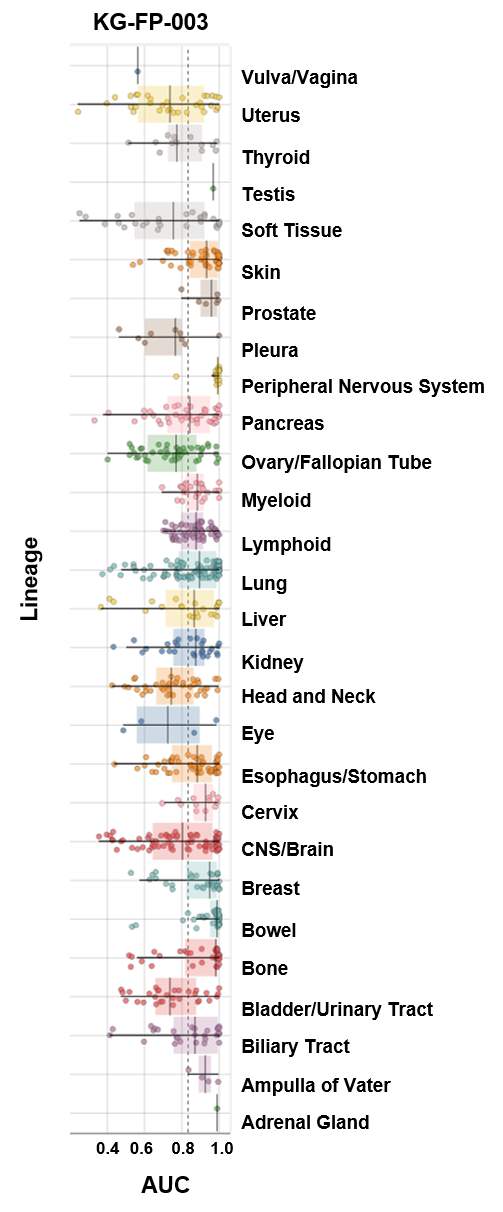


**Fig. S15 Lineage enrichment analysis of PRISM data.** Boxplots represent the AUC values of each cell line within respective (sub)lineages. The dashed line indicates the average AUC across all cell lines.

**
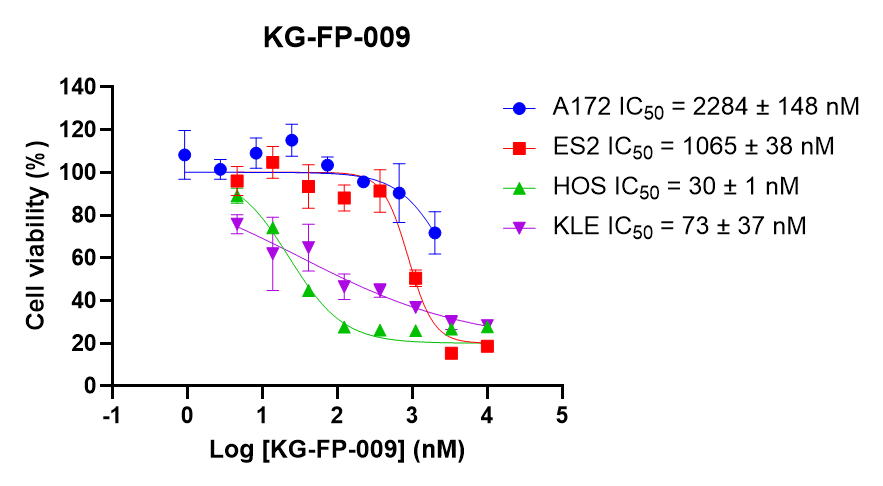
**

**Fig. S16** Cell viability of different cell lines following 72-hour treatment with KG-FP-009 at indicated concentrations. Viability was measured using a standard CTG assay, and Data are representative of three independent experiments. Data are representative of three independent experiments.


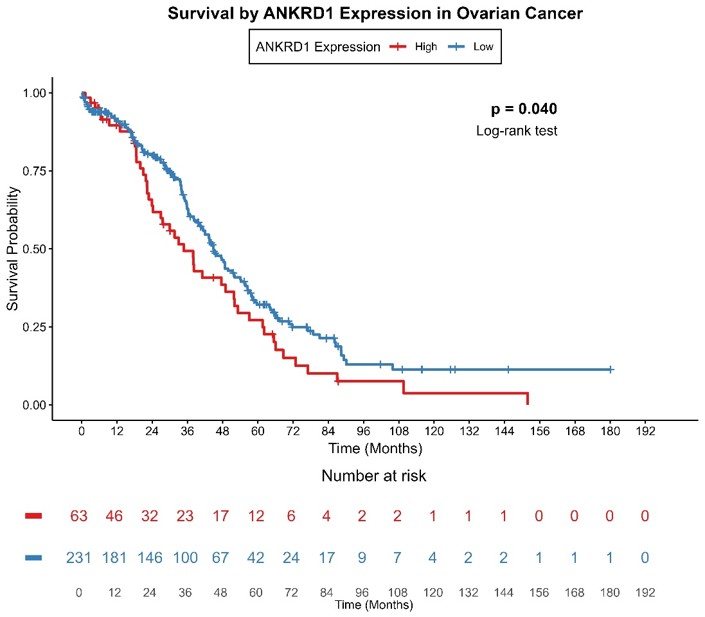
**Fig. S17** Analysis of transcriptomic data from the TCGA database demonstrated that elevated ANKRD1 expression significantly correlated with worse overall survival (OS) in the TCGA-OV cohort (p < 0.05).

**Fig. S18** Analysis of transcriptomic data from the TCGA database demonstrated that elevated *CCN1* (CYR61) expression significantly correlated with worse overall survival (OS) in the TCGA-OV cohort (p < 0.05).


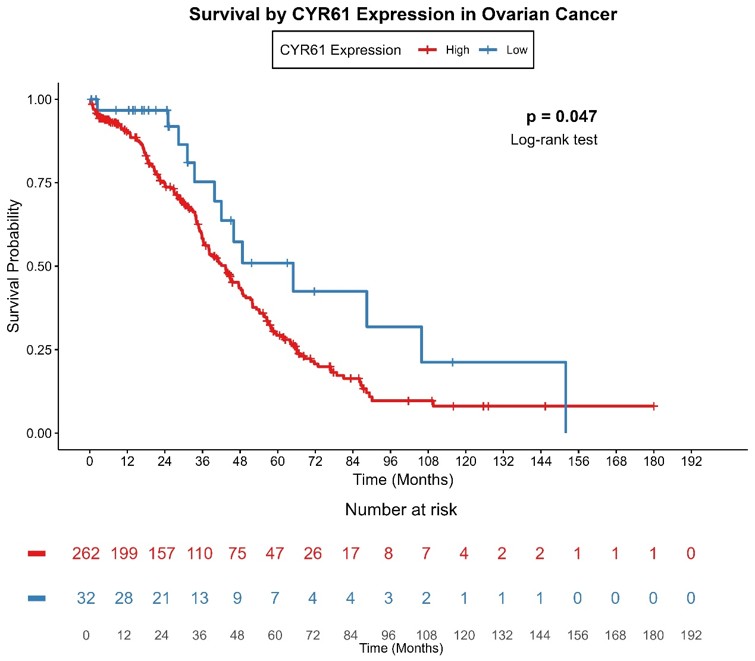


**Fig. S19** Immunoblot analysis in ES2 cells demonstrated a dose-dependent reduction of TEAD1 following 24 h treatment with KG-FP-003
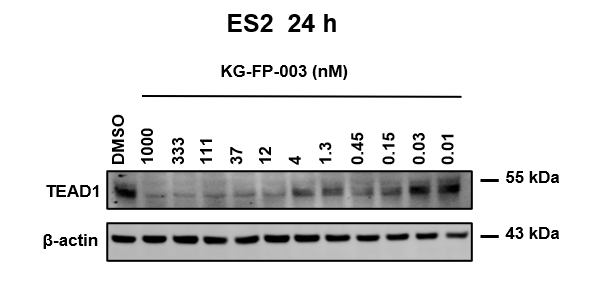
.

**
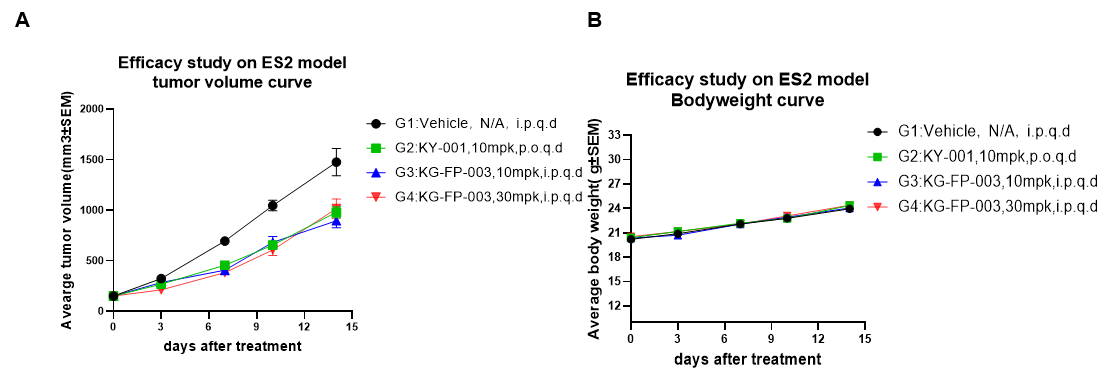
**

**Fig. S20** Comprehensive evaluation of antitumor efficacy in the ES-2 xenograft mouse model. **(A)** Tumor growth curves of ES-2 xenograft-bearing BALB/c nude mice treated with vehicle (DMSO), KY-001, or KG-FP-003 (n = 6 per group). **(B)** Body weight monitoring of mice throughout the treatment period to assess tolerability.

**Fig. S21** DIA-based proteomic analysis of TEAD1 expression regulation in A172 cells treated with 100 nM KG-FP-003 for 24 hours.


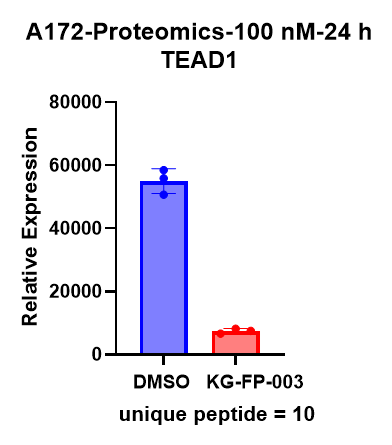


**
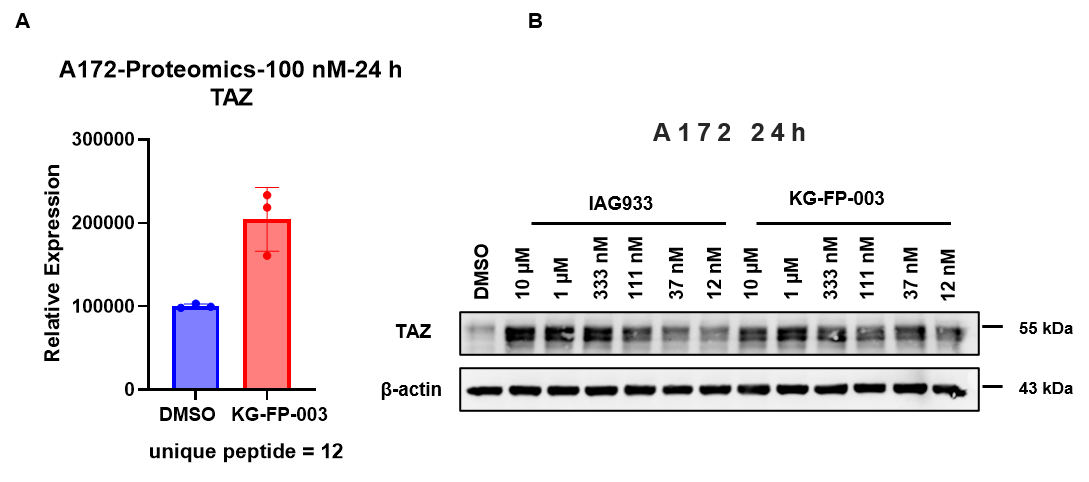
**

**Fig. S22 (A)** DIA-based proteomic analysis showing regulation of TAZ expression in A172 cells treated with 100 nM KG-FP-003 for 24 hours. **(B)** Immunoblot validation confirms that KG-FP-003 and IAG933 increase TAZ protein levels in A172 cells.

**
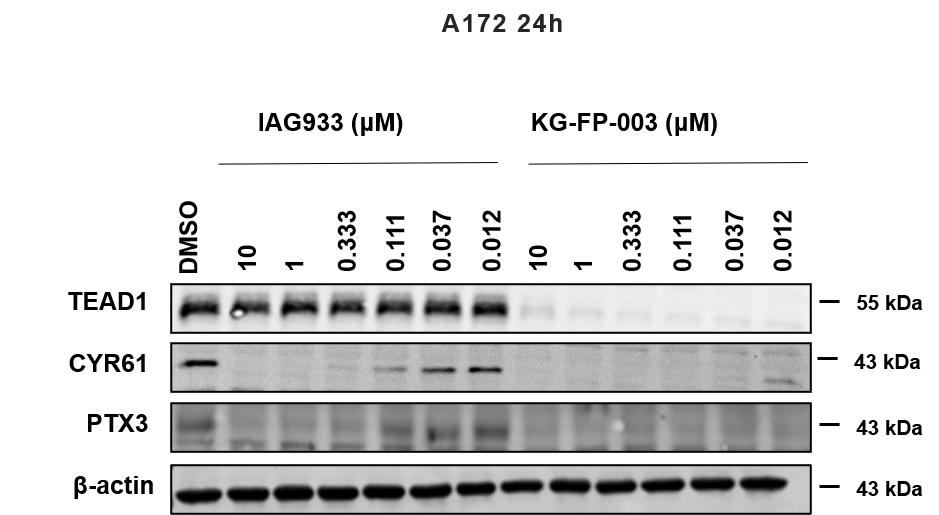
**

**Fig. S23** Immunoblot analysis showing dose-dependent changes in TEAD target gene expression in A172 cells treated with KG-FP-003 and IAG933 for 24 hours.


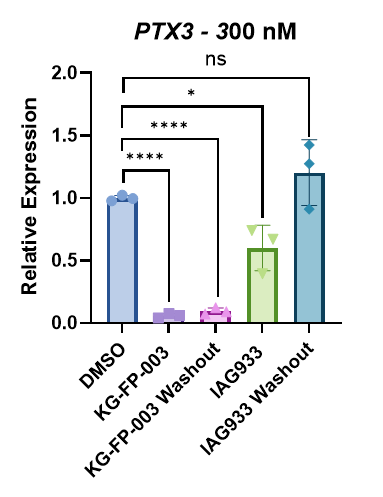


**Fig. S24** The impact of KG-FP-003 and IAG933 on *PTX3* expression in A172 cells. For washout experiments, cells were pretreated with IAG933 or KG-FP-003 for 6 hours, then washed three times and incubated for an additional 18 hours before sample collection. Data are representative of three independent experiments.


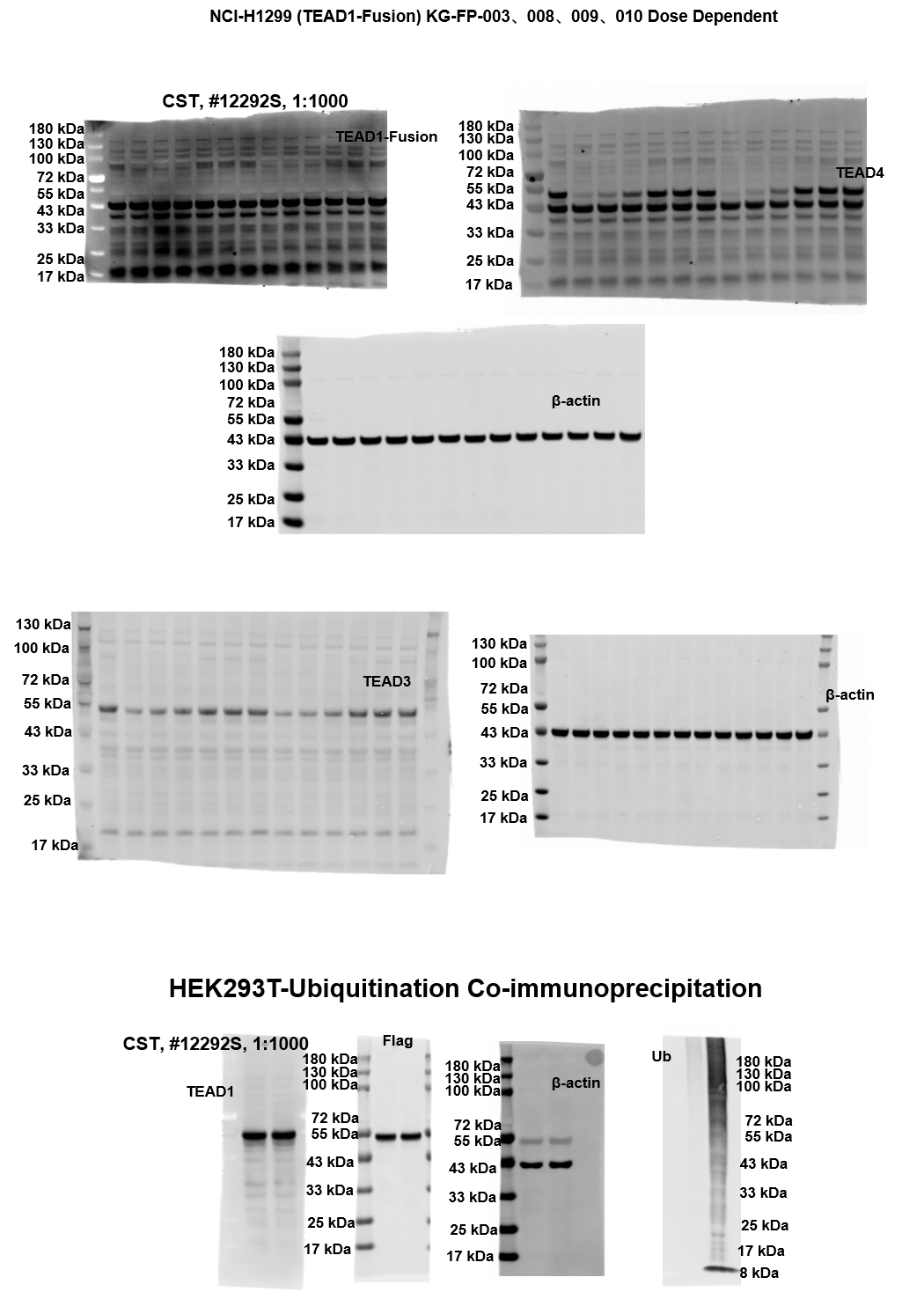
**Fig. S****25** Uncropped immunoblots. Corresponds to Figure 1 in the main text.


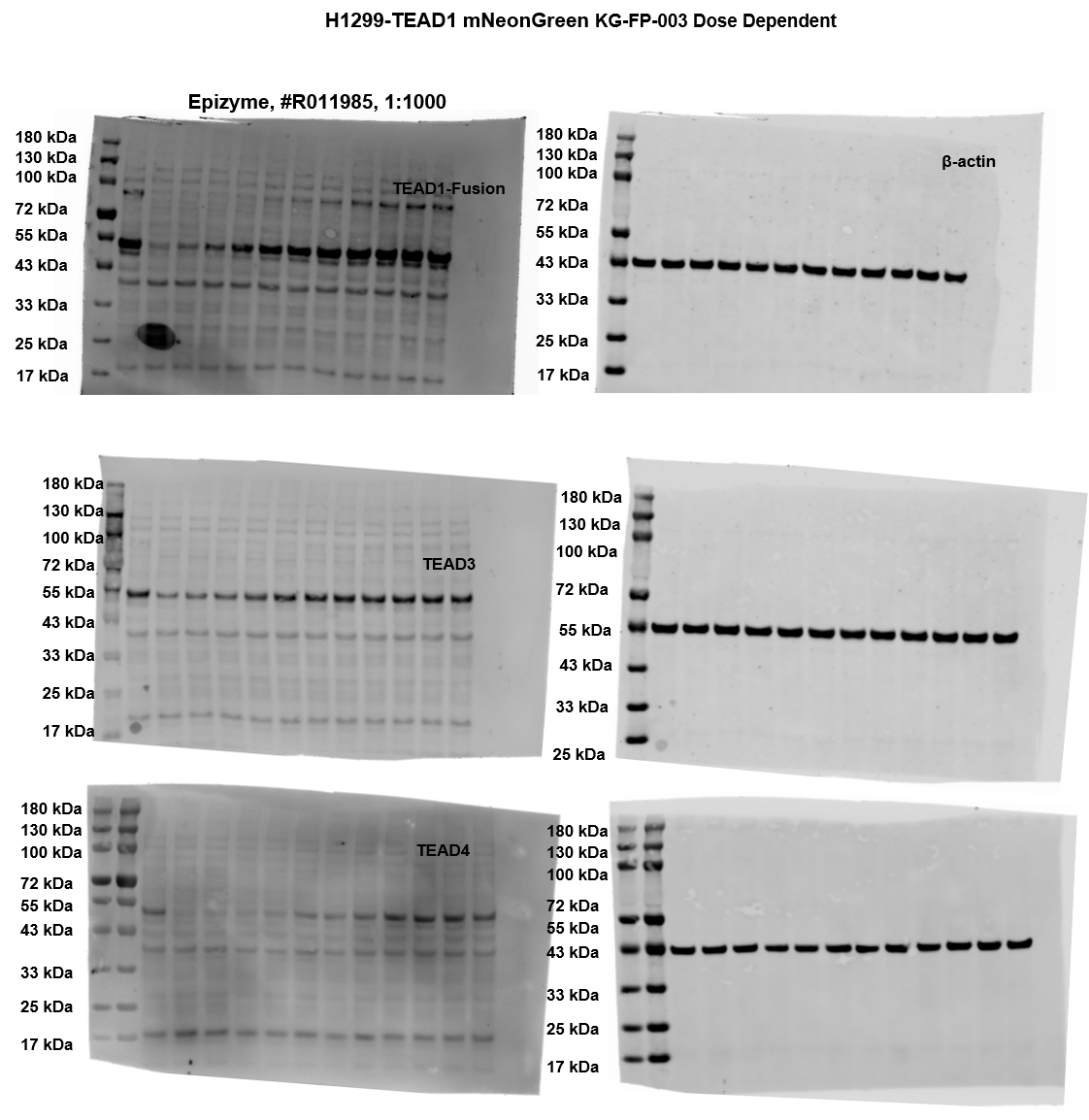


**Fig. S26** Uncropped immunoblots. Corresponds to Figure 2 in the main text.


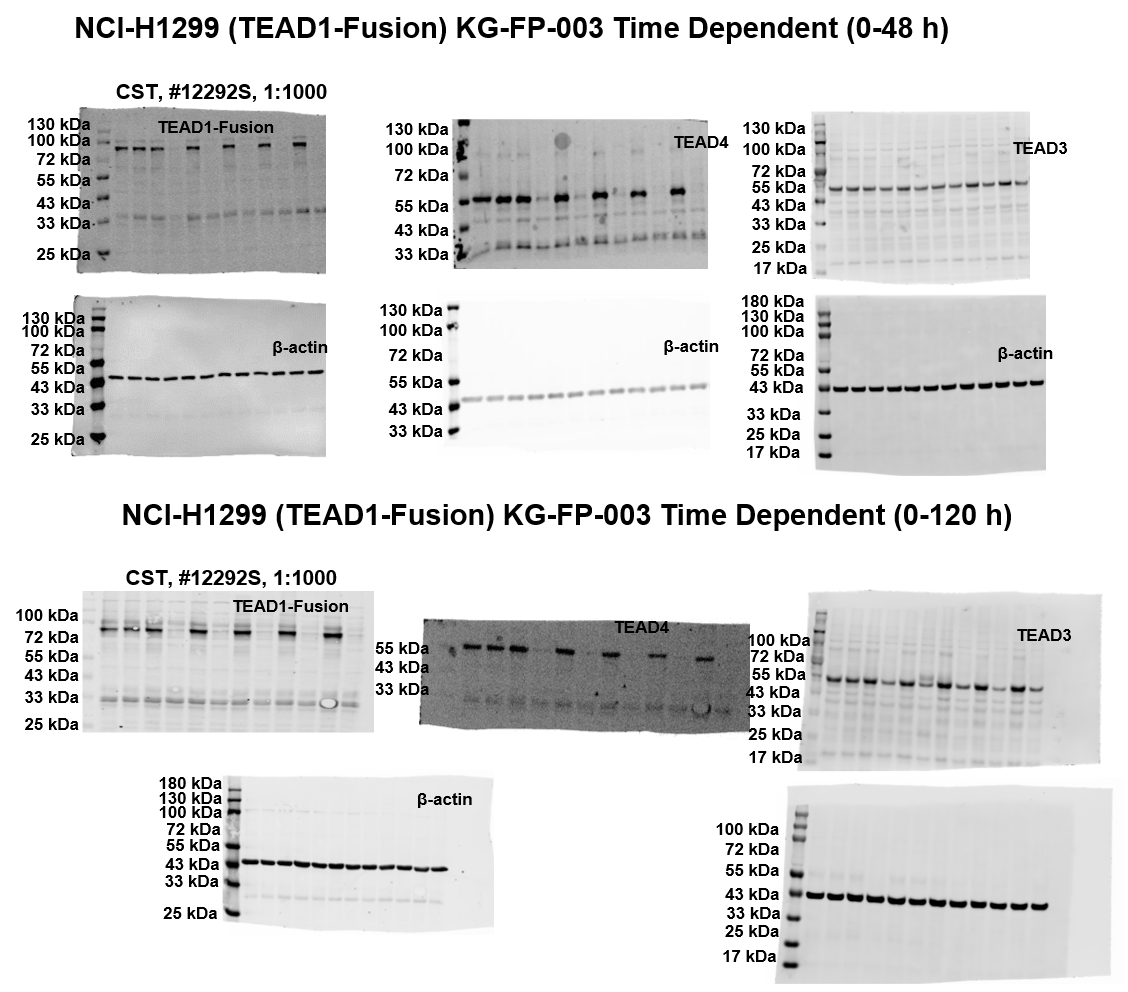


**Fig. S27** Uncropped immunoblots. Corresponds to Figure 2 in the main text.


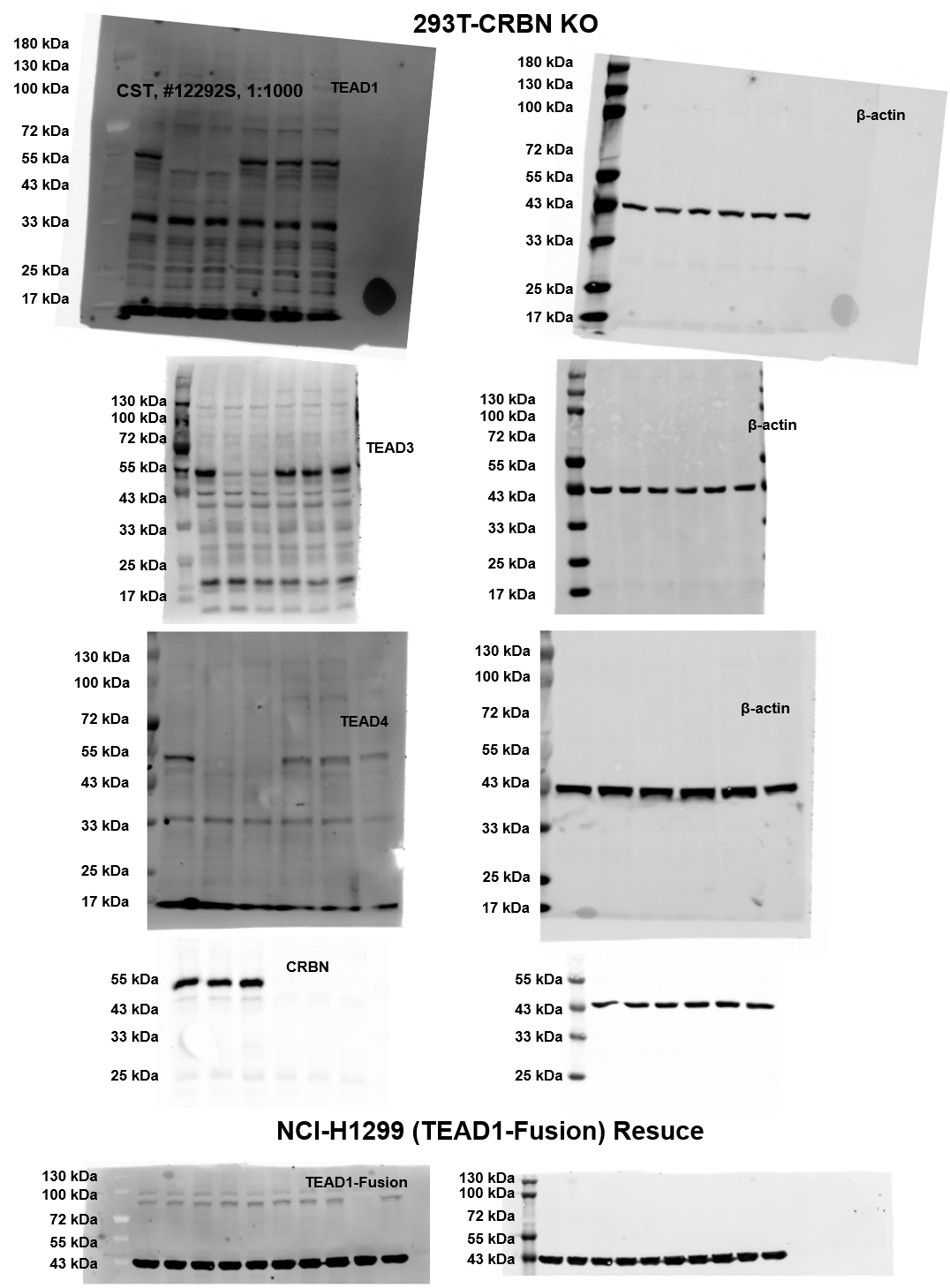


**Fig. S28** Uncropped immunoblots. Corresponds to Figure 2 in the main text.

**
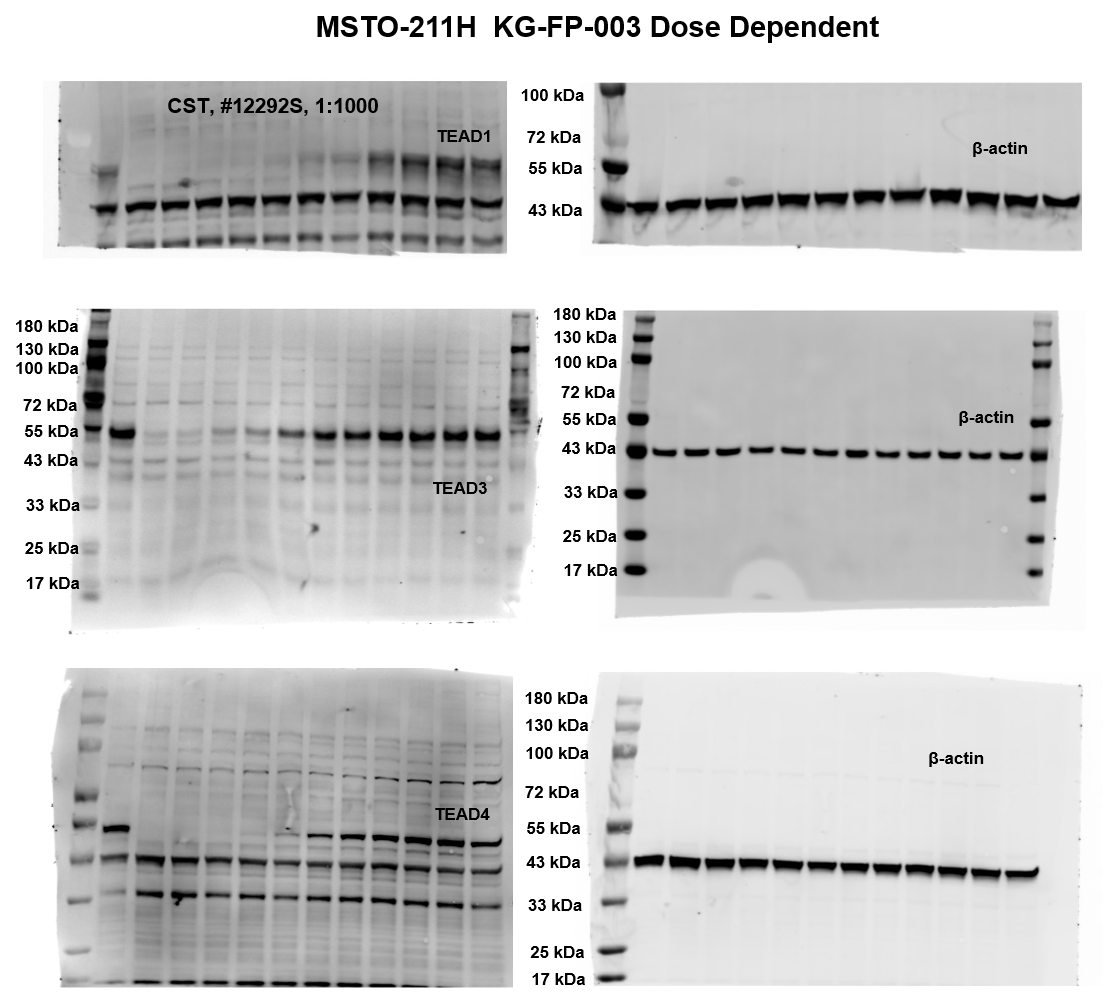
**

**Fig. S29** Uncropped immunoblots. Corresponds to Figure 3 in the main text.

**
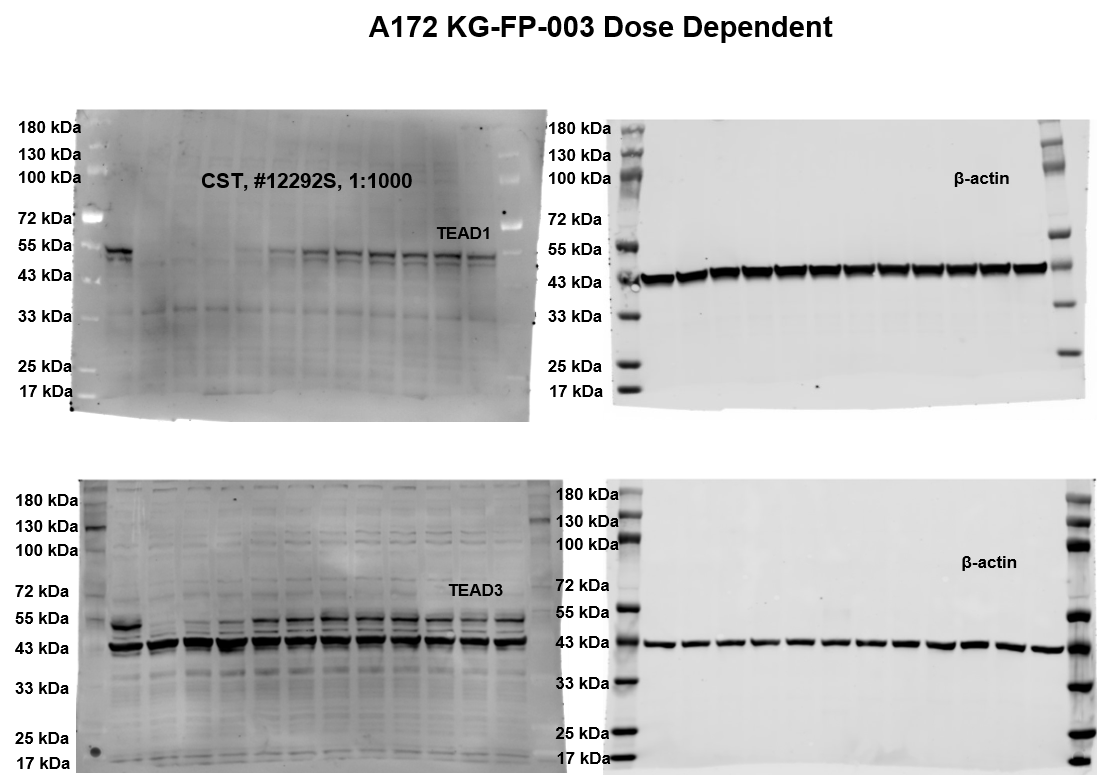
**

**Fig. S30** Uncropped immunoblots. Corresponds to Figure 5 in the main text.

**
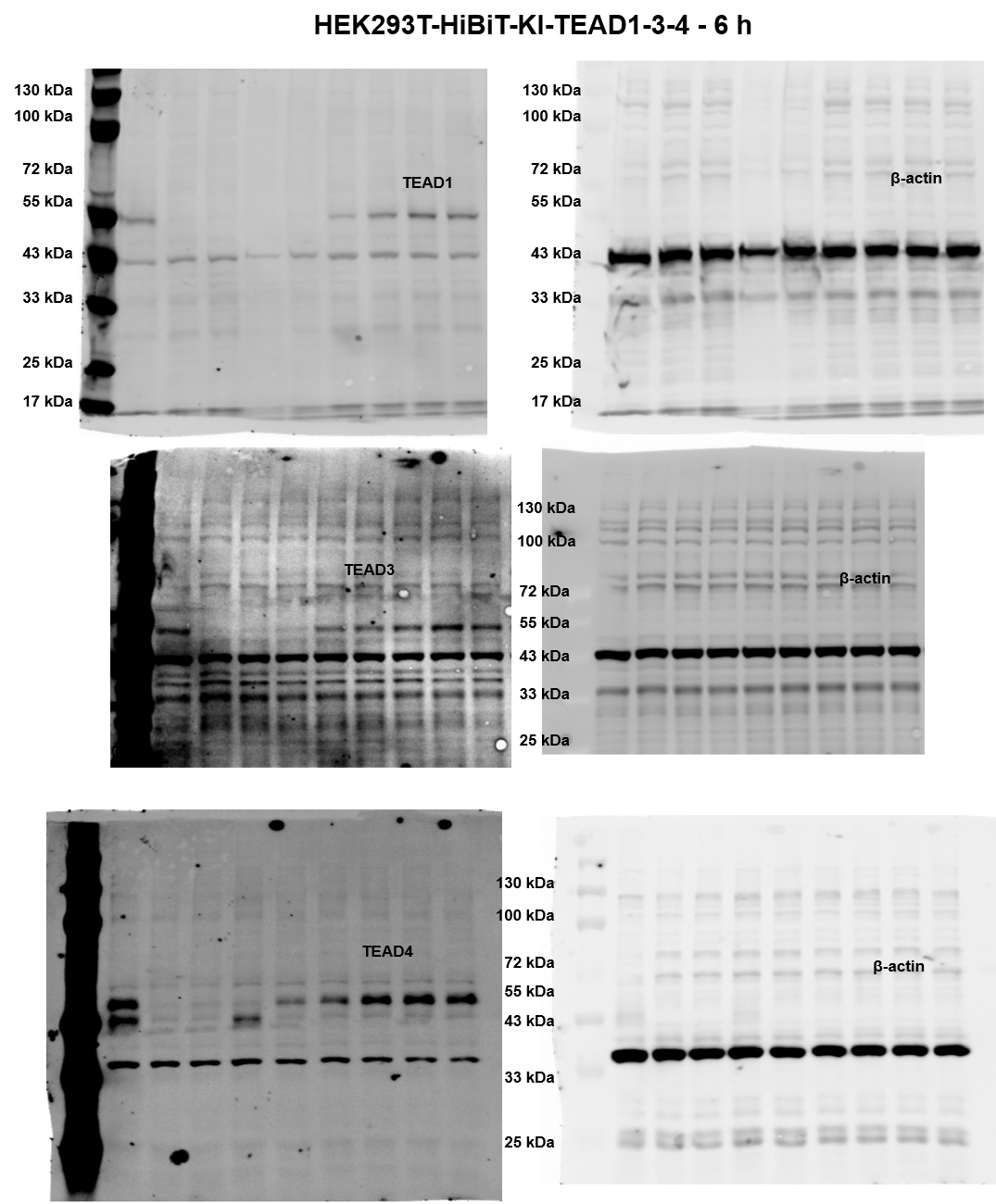
**

**Fig. S31** Uncropped immunoblots. Corresponds to Figure S4 in the main text.

**
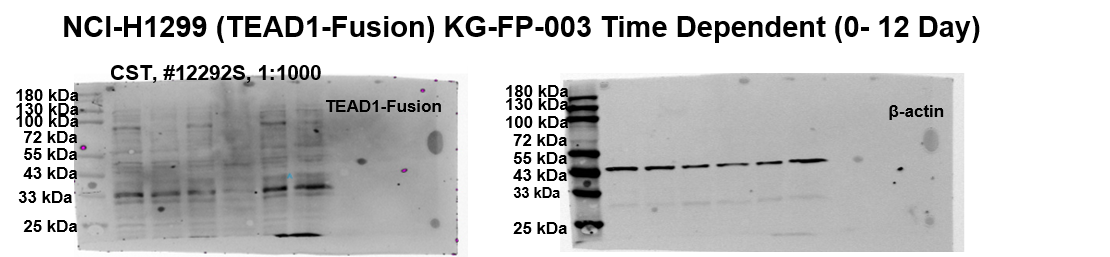
**

**Fig. S32** Uncropped immunoblots. Corresponds to Figure S6 in the main text.

**
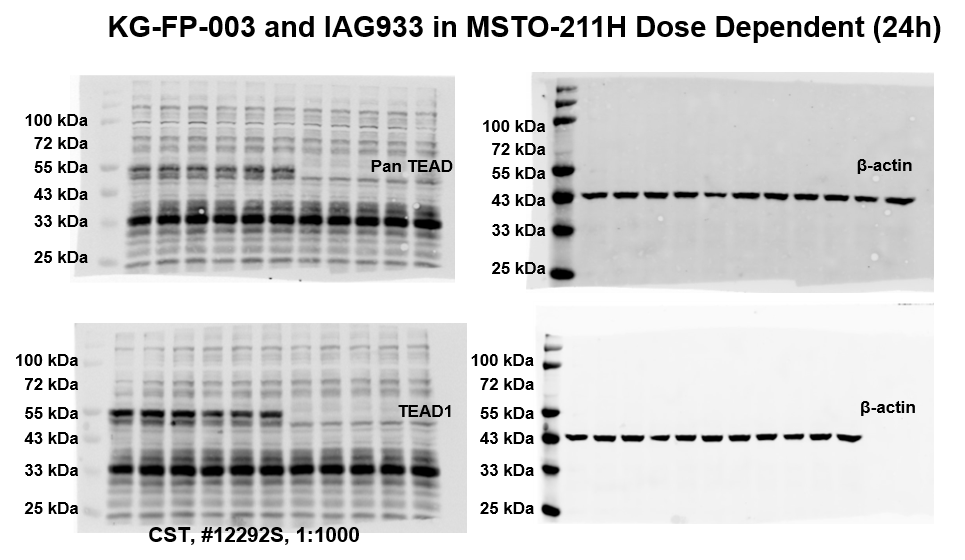
**

**Fig. S33** Uncropped immunoblots. Corresponds to Figure S7 in the main text.

**
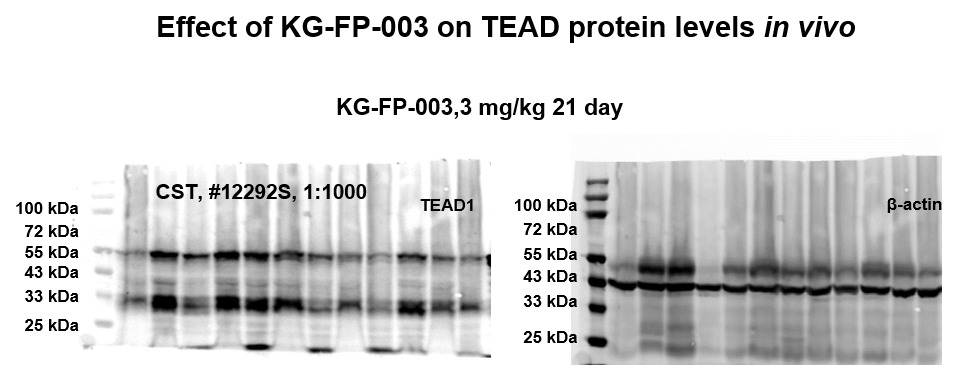
**

**Fig. S34** Uncropped immunoblots. Corresponds to Figure S10 in the main text.

**
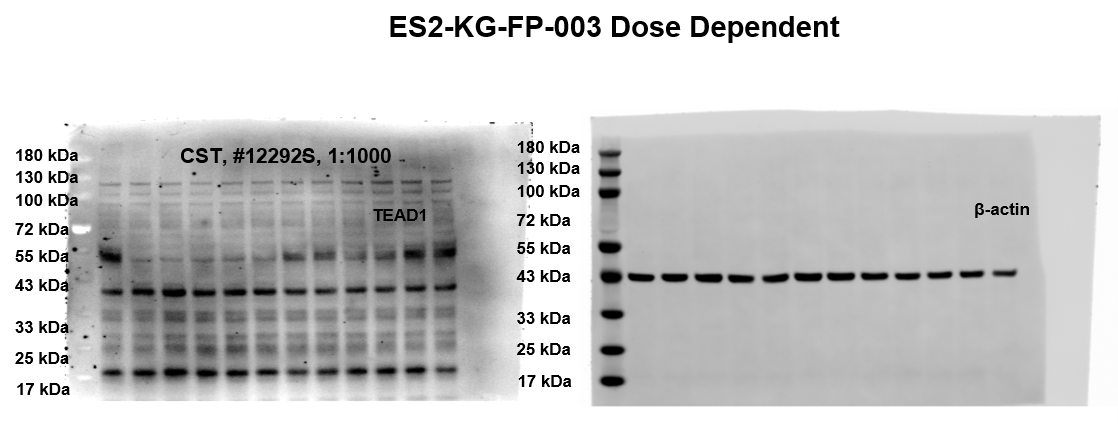
Fig. S35** Uncropped immunoblots. Corresponds to Figure S19 in the main text.

**Fig. S36** Uncropped immunoblots. Corresponds to Figure S22 in the main text.

**
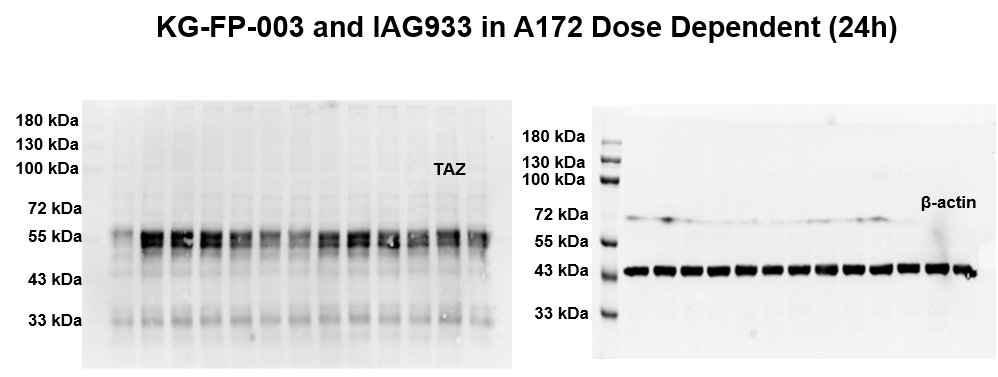
**

**
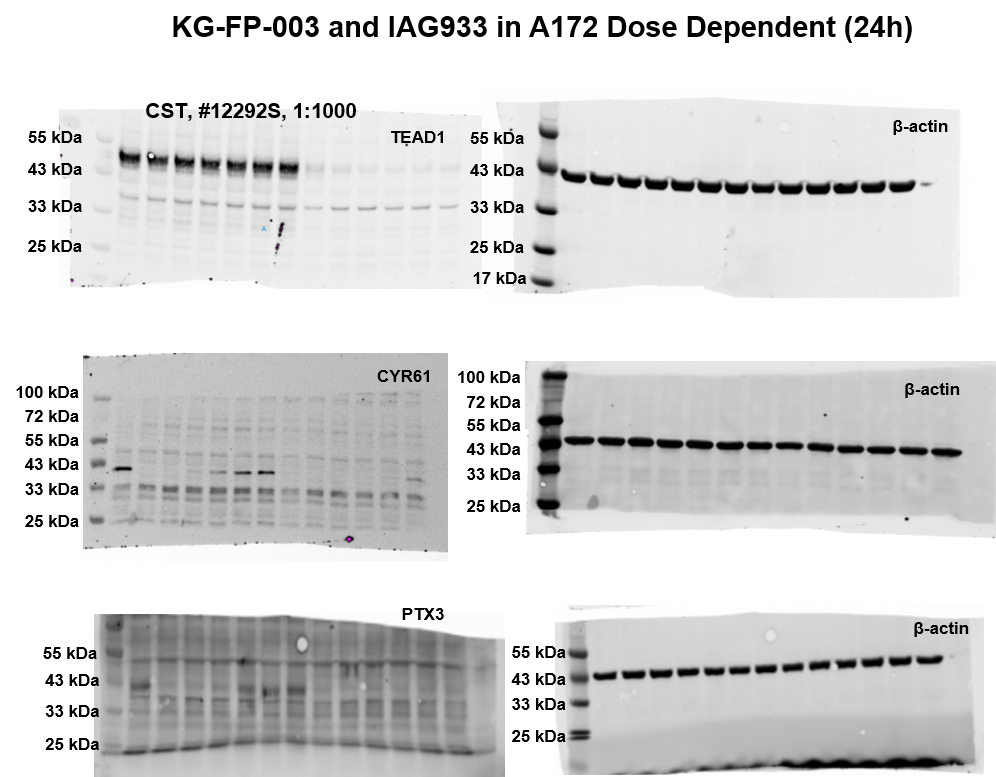
**

**Fig. S37** Uncropped immunoblots. Corresponds to Figure S23 in the main text.

**^1^H-HMR spectrum of target compounds**

**Fig. S38** ^1^H-NMR spectrum of compound KG-FP-003.

**Fig. S39** ^1^H-NMR spectrum of compound KG-FP-008.

**Fig. S40** ^1^H-NMR spectrum of compound KG-FP-009.

**Fig. S41** ^1^H-NMR spectrum of compound KG-FP-010.

|  | **Forward (5' - 3')** | **Reverse (5' - 3')** |
| --- | --- | --- |
| ***CTGF*** | GCGAGGAGTGGGTGTGTGAC | ACCAGGCAGTTGGCTCTAATCATAG |
| ***BMF*** | CCCACCAGCCAGGAAGACAAAG | AGGAAGCCGATAGCCAGCATTG |
| ***ANKRD1*** | CAACGCCAAAGACAGAGAAGGAG | GCCATACATAATCAGGAGTCGGATC |
| ***NPPB*** | CGCTCCTGCTCCTGCTCTTC | TTCCAAGTCCGAGGCTGAACC |
| ***PTX3*** | GGCGGCTACCACTGTTGAGATG | CCACCCACACAGCAGCCATTC |
| ***GAPDH*** | ACCCACTCCTCCACCTTTGAC | TCCACCACCCTGTTGCTGTAG |

**Table. S1** Primer sequences used for RT-qPCR analysis
